# Distribution and evolution of glycoside hydrolase family 45 cellulases in nematodes and fungi

**DOI:** 10.1186/1471-2148-14-69

**Published:** 2014-04-01

**Authors:** Juan E Palomares-Rius, Yuuri Hirooka, Isheng J Tsai, Hayato Masuya, Akina Hino, Natsumi Kanzaki, John T Jones, Taisei Kikuchi

**Affiliations:** 1 Division of Parasitology, Faculty of Medicine, University of Miyazaki, Miyazaki 889-1692, Japan; 2 Instituto de Agricultura Sostenible (IAS), Consejo Superior de Investigaciones Científicas (CSIC), Campus de Excelencia Internacional, Apdo. 4084, 14080 Córdoba, Spain; 3 Forestry and Forest Products Research Institute, Tsukuba, Ibaraki 305-8687, Japan; 4 Biodiversity (Mycology), Eastern Cereal and Oilseed Research Centre, Agriculture and Agri-Food Canada, Ottawa, ON K1A0C6, Canada; 5 James Hutton Institute, Invergowrie, Dundee DD2 5DA, UK; 6 Biology Department, Ghent University, K.L. Ledeganckstraat 35, 9000 Ghent, Belgium

**Keywords:** Bursaphelenchus, Cellulases, Horizontal gene transfer, Ascomycota, Fungi

## Abstract

**Background:**

Horizontal gene transfer (HGT) has been suggested as the mechanism by which various plant parasitic nematode species have obtained genes important in parasitism. In particular, cellulase genes have been acquired by plant parasitic nematodes that allow them to digest plant cell walls. Unlike the typical glycoside hydrolase (GH) family 5 cellulase genes which are found in several nematode species from the order Tylenchida, members of the GH45 cellulase have only been identified in a cluster including the families Parasitaphelenchidae (with the pinewood nematode *Bursaphelenchus xylophilus*) and Aphelenchoididae, and their origins remain unknown.

**Results:**

In order to investigate the distribution and evolution of GH45 cellulase genes in nematodes and fungi we performed a wide ranging screen for novel putative GH45 sequences. This revealed that the sequences are widespread mainly in Ascomycetous fungi and have so far been found in a single major nematode lineage. Close relationships between the sequences from nematodes and fungi were found through our phylogenetic analyses. An intron position is shared by sequences from *Bursaphelenchus* nematodes and several Ascomycetous fungal species.

**Conclusions:**

The close phylogenetic relationships and conserved gene structure between the sequences from nematodes and fungi strongly supports the hypothesis that nematode GH45 cellulase genes were acquired via HGT from fungi. The rapid duplication and turnover of these genes within *Bursaphelenchus* genomes demonstrate that useful sequences acquired via HGT can become established in the genomes of recipient organisms and may open novel niches for these organisms to exploit.

## Background

Cellulose, a polymer of β-1,4-linked glucose molecules, is the major polysaccharide component of plant cell walls and is the most abundant organic polymer on Earth. Many microorganisms produce cellulases to degrade cellulose in order to use it as a carbon source. For plant pathogens, the plant cell wall is the primary barrier that they need to overcome and the production of enzymes capable of degrading cellulose is therefore of critical importance for colonization of plants.

Most animals (Metazoa) do not have endogenous cellulases and rely instead on intestinal symbiotic microorganisms for cellulose digestion. However, recent studies have shown that some insects and plant-parasitic nematodes have endogenous cellulases that degrade cellulose polymers [1,2].

Cellulases can be grouped into families based on their sequence and on the basis of hydrophobic cluster analysis [3]. Fourteen families of glycosyl hydrolases (GH) are known to include proteins that degrade cellulose (http://www.cazy.org). It is thought that proteins within each group are structurally related and are likely to have evolved from a common ancestor [4].

Cellulases from two distinct glycosyl hydrolase families (GH5 and GH45) have been found in nematodes. GH5 cellulases have been found in a wide range of Clade 12 Tylenchid plant parasitic nematodes and show relatively high similarity to bacterial GH5 sequences, leading to the suggestion that they were acquired *via* horizontal gene transfer (HGT) from bacteria [5-7]. However, another plant-parasitic nematode *Bursaphelenchus xylophilus*, which is located in Clade 10 as described by van Megen et al. [8] and is not directly related to the Clade 12 Tylenchid plant parasites, has GH45 cellulases rather than GH5 [9]. These two GH families show little amino acid similarity to each other and have distinct kinetic mechanisms, catalytic residues and three dimensional structures although both catalyze the breakdown of similar substrate; cellulose and hemicelluloses [10,11].

The origin of the nematode GH45 cellulases remains unclear, although HGT from fungi seems likely given the high similarity to fungal GH45 cellulases and the absence of sequences resembling GH45 cellulases from all other nematodes analysed to date.

*Bursaphelenchus xylophilus* is the causal agent of pine wilt disease [12]. In their pathogenic life cycle the nematode is transmitted from trees killed by pine-wilt to healthy pines by vector beetles. Once the nematodes enter the tree, they feed on plant cells in the tree, leading disruption of pine tissues and lethal wilt. As the pine wilts and dies, the nematodes start to feed on fungi that invade the dying tree. Furthermore, most *Bursaphelenchus* species are solely fungal feeders and all species rely on fungi as a food source at some stage of their life cycle.

In this study we have conducted a wide ranging screen and intensive phylogenetic analysis of GH45-like sequences in nematodes and fungi, particularly those found in association with plants. Our results show a wide distribution of GH45 cellulases in Ascomycetous fungi and a narrow but concentrated distribution in nematodes, a single lineage that includes a number of facultative plant parasites. The close relationships between the nematode and fungal sequences, as well as a shared intron position in some of the nematode and fungal sequences, suggest that nematode GH45 cellulases were acquired via HGT from fungi and subsequently underwent repeated duplication within nematode genomes.

## Results

### Amplification of GH45 cellulase sequences

Genomic DNA was extracted from 289 fungal species/strains and 26 nematode species/strains (Additional file 1: Table S1, S2) and used for PCR amplification with a degenerate primer pair designed from a conserved region of known GH45 cellulases (Additional file 2: Figure S1). Clear bands were observed at around 500–2000 bp following agarose gel electrophoresis. The majority of successful amplifications were from Ascomycetous fungi or *Bursaphelenchus* nematode species. No amplification was seen with Basidiomycetous fungi or distantly related (Clade 12) nematode species including *Aphelenchus avenae* and *Pratylenchus* sp., suggesting the absence of GH45 in these species.

Sequence analysis revealed most of those fragments contained the conserved sequence of GH45 cellulases including two catalytic core residues (Asp, Asp) (Additional file 2: Figure S1). In total we obtained 47 sequences from 13 nematode species (out of 20 species tested) including *B. doui*, *B. conicaudatus*, *B. purvispicularis*, *B. xylophilus*, *B. mucronatus*, *B. luxuriosiae*, *B. okinawaensis*, *B. kiyoharai*, *Bursaphelenchus* sp1, *Bursaphelenchus* sp2, *B. yongensis* and *B. poligraphi* (Additional file 1: Table S3). *Ruehmaphelenchus* sp. in this study and *Aphelenchoides besseyi*[13] in a previous study are the only species other than *Bursaphelenchus* species from which GH45-like sequences were identified. For fungi 70 GH45-like sequences were identified from 61 fungal species (out of 259 species/strains tested), all of which belong to Ascomycota (Additional file 1: Table S4).

### Gene structures

**Figure 1:**
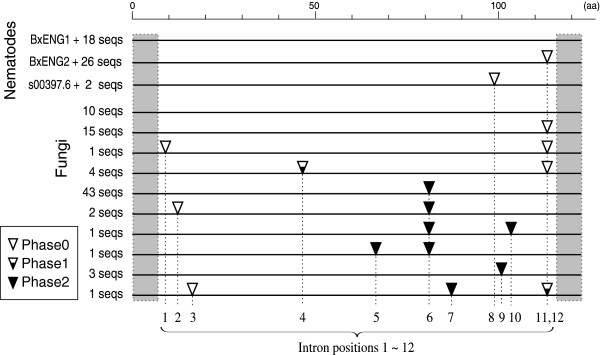
**Intron/exon structures of GH45 fragments of nematodes and fungi.** Intron positions found in nematode and fungal sequences are shown by triangles on a simplified amino acid alignment of GH45 proteins (the original alignment for some specific species can be found in Additional file 2: Figure S1). Conserved regions used to design primers are box shaded. Phase of the introns is shown by distinct triangles. Thirteen intron positions were found in total from nematodes and fungi and position 11 is shared by fungi and nematode sequences.

The nematode GH45 sequences can be broadly grouped into two different intron-types, some of which were successfully confirmed by RT-PCR or RNA-seq data (Additional file 1: Table S3): genes with no intron (p0) and genes with one intron at position 11 (P11) (Figure 1, Additional file 1: Table S3). Position 11 introns in the nematodes varied in length from 36 bp to 220 bp (Additional file 1: Table S3). Some nematode species contained more than one GH45 sequence and included both P0 and P11 intron-types. These species included *B. xylophilus*, *B. doui*, *B. purviscularis*, *B. mucronatus* and *B. luxuriosae*. We were only able to amplify one intron-type from other species: *B. conicaudatus*, *B. kiyoharai* and *Bursaphelenchus* sp1 contained only GH45 sequences with P11 introns while *Bursaphelenchus* sp3*, B. okinawaensis*, *B. yongensis*, *B. poligraphi* and *Bursaphelenchus* sp2 contained only p0 GH45 sequences. Only intronless sequences were identified from *Ruehmaphelenchus* sp.

Eleven GH45 genes were predicted in the recently published *B. xylophilus* genome sequence [14]. Six of these 11 genes have no intron (p0), 2 genes have introns at position 11 (p11) and 3 genes at position 8 (p8) (Figure 1, Additional file 1: Table S3).

Eleven intron positions were found in the fungal sequences. Most of the fragments contained only one intron (Figure 1). Forty-three sequences had one intron at position 6, 15 sequences had one intron at position 11 and 10 had no intron in the amplified fragment. The intron lengths were 46 bp to 343 bp. One intron position (p11) was shared by the nematodes and several fungal species.

### Phylogenetic analysis

#### Nematode sequences

**Figure 2:**
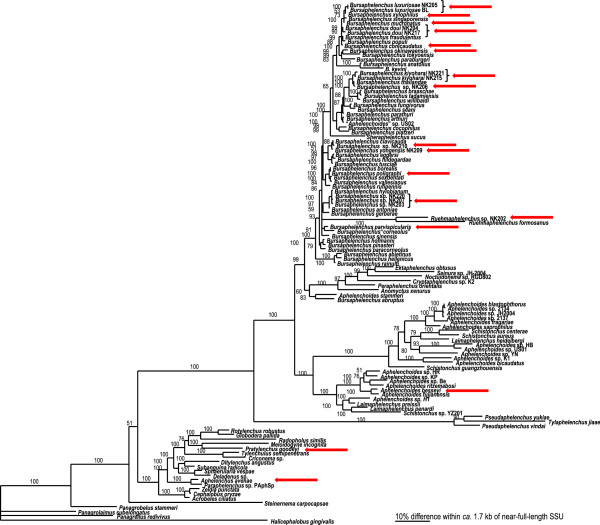
**The molecular phylogenetic relationships among selected species of Aphelenchoidea, Tylenchoidea and Cephaloboidea.** 10001st Bayesian tree inferred from near-full-length small subunit of ribosomal RNA gene under GTR + I + G model. The figure has been adapted from the phylogenetic tree provided by Kanzaki & Tanaka [15]. Arrows indicate species used in this study.

To reconstruct the evolutionary relationship between the species with successful amplified GH45 sequences, a phylogenetic tree was built based on nearly full length 18S ribosomal RNA (rRNA) gene from these species. A comprehensive list of nematodes including the superfamily Aphelenchoidea, the order Tylenchida and some members of Cephaloboidea were used for the analysis. However, a major effort in sequences for phylogenetic reconstruction was done for members of Aphelenchoidea, which harbour GH45 sequences (Figure 2).

The phylogeny of the *Bursaphelenchus* genus is characterised by a well defined clade (100 PP) which includes *B. luxuriosae*, *B. xylophilus*, *B. mucronatus*, *B. doui*, *B. conicaudatus,* and will be referred as the “*xylophilus* group” throughout the manuscript. *B. kiyoharai* and *Bursaphelenchus* sp1 are clustered into a clade next to that of the *xylophilus* group (100 PP). The rest of the species considered in the study are distributed throughout the different subclades formed.

**Figure 3:**
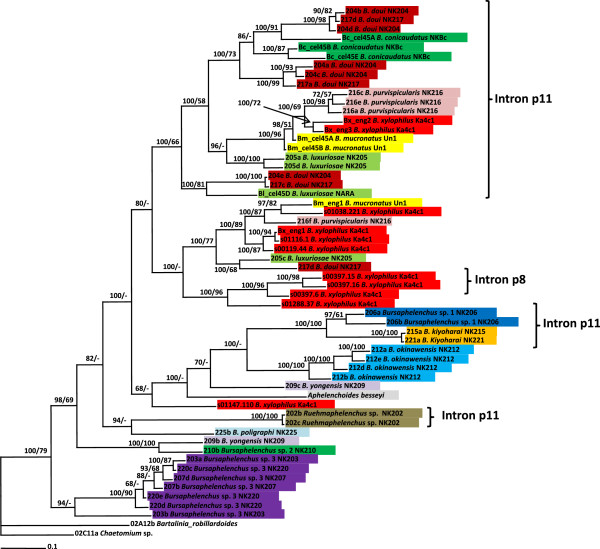
**Bayesian 50% majority rule consensus tree of Aphelenchoidea with GH45 nucleotide sequences under 010234 + I + G + F model.** Posterior probabilities more than 65% are given for appropriate clades; bootstrap values greater than 50% are given on appropriate clades in ML analysis.

Phylogenetic trees of nematode GH45 nucleotide sequences are shown in Figure 3 with two fungal GH45 sequences included as outgroup. Similar topology was shown using amino acid sequences (Additional file 2: Figure S2). The phylogeny revealed that the *xylophilus* group clustered into one large clade comprised by two well supported subclades: subclade 1 is comprised by sequences with intron 11 (p11) and subclade 2 with sequences that show other intron positions (p0 or p8). Sequences from *B. okinawaensis* are clustered together with those from *B. kiyoharai* and *Bursaphelenchus* sp1 which comprise a sister clade of the *xylophilus* group in the SSU tree. *Bursaphelenchus* sp3 sequences were clustered into an independent clade at the basal position. The sequences from *Ruehmaphelenchus* sp. were positioned inside the *Bursaphelenchus* sequences with a long branch; a similar pattern is also observed in the SSU tree.

Ten out of 11 *B. xylophilus* GH45 genes from the genome sequence of this species were clustered into four clades in the aforementioned large clade. These clades were composed of the following sequences: i) Bx-eng-2 and Bx-eng-3 with *B. purvispicularis* and *B. mucronatus* sequences; ii) s01038.221, Bx-eng-1, s01116.1 and s00119.44 with *B. purvispicularis* and *B. mucronatus* sequences; iii) s00397.15, s00397.16, s00397.6 and s01288.37 sequences and iv) s01147.110 sequence occupying a basal position with other *Bursaphelenchus* species sequences and *A. besseyi*. This close relationship between sequences for the same species is also observed for other species with the exceptions of *B. yongensis* and *B. poligraphi* from which we were able to identify only one and two GH45 sequences respectively.

The positions of the *B. purvispicularis* GH45 sequences differed greatly from the position of this species within the SSU phylogeny. The *A. besseyi* GH45 sequence [13] was nested within the sequences from the *Bursaphelenchus* species.

#### Fungal sequences

**Figure 4:**
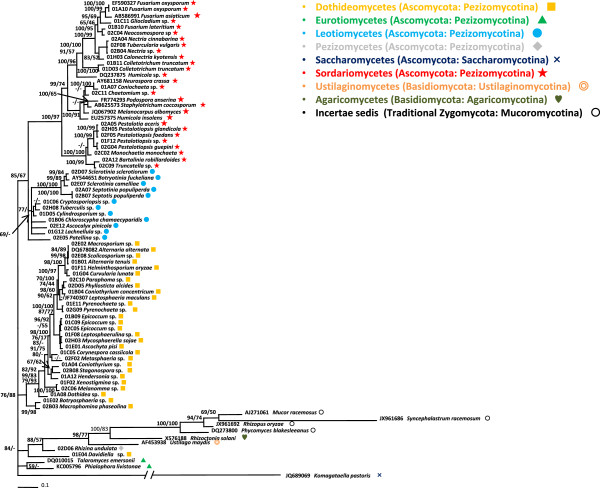
**Bayesian 50% majority rule consensus tree of fungi with partial LSU subunit of ribosomal RNA gene under TIM3 + G model.** Ascomycota, Basidiomycota and traditional Zygomycota were included in the tree. Posterior probabilities more than 65% are given for appropriate clades; bootstrap values greater than 50% are given on appropriate clades in ML analysis.

A phylogenetic tree based on the large subunit ribosomal RNA genes (LSU) of fungi is shown in Figure 4. A comprehensive set of species belonging to Ascomycota ranging from Sordariomycetes to Saccharomycetes were included in the phylogenetic tree. The topology of the tree correlated well with previously described phylogenetic data from fungi, even though we only sequenced a single locus and some groups were not represented by a large number of species e.g. [16-18]. The Sordariomycetes clade is well supported with 100 PP and 97 BS values. Leotiomycetes and Dothideomycetes were nearly monophyletic although *Patellina* sp. (02E05) did not nest in the main clade. Two species of Eurotiomycetes were grouped and made a distinct clade with low phylogenetic values. The other representative species of each class *e.g*., Pezizomycetes, Basidiomycetes and Agaricomycetes, were supported by high phylogenetic values and long branches. The fact that these species did not taxonomically correlate with previous phylogenetic data was most likely because we used only a single locus in this study.

**Figure 5:**
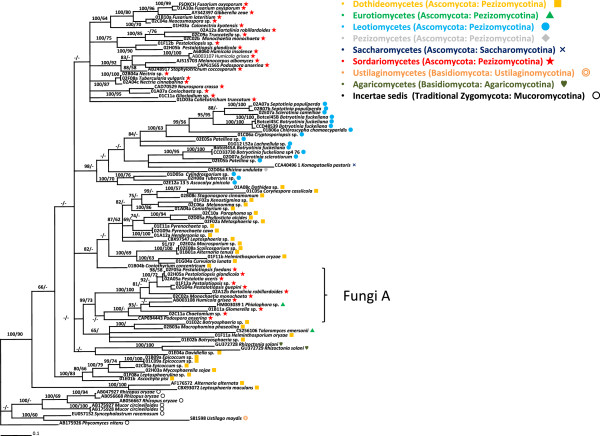
**Bayesian 50% majority rule consensus tree of fungi with GH45 nucleotide sequences under TVM + I + G model.** Ascomycota, Basidiomycota and traditional Zygomycota were included in the tree. Posterior probabilities more than 65% are given for appropriate clades; bootstrap values greater than 50% are given on appropriate clades in ML analysis.

A phylogenetic tree of GH45 sequences based on nucleotide sequences is shown in Figure 5 and the phylogenetic tree based on amino acid sequences is shown in Additional file 2: Figure S3. The trees of LSU and GH45 sequences were not completely consistent for some species, genera and classes, but showed a good consistency in general. In the phylogenetic trees of GH45 sequences, none of the classes we analysed were monophyletic, except for Agaricomycetes. Leotiomycetes was nearly monophyletic as *Komagataella pastoris* and *Rhizina undulata* were clustered with the Leotiomycetes clade in the phylogenetic trees of GH45 sequences. This class is also nearly clustered as monophyletic in the phylogenetic trees of amino acid sequences. Sordariomycetes were divided into two clades in the both phylogenetic trees. Interestingly, the two clades showed several genera or species duplications such as *Pestalotiopsis glandicola*, *Bartalinia robillardoides*, *Monochaetia monochaeta* and *Humicola grisea*. Dothideomycetes was divided into several clades that were not sorted by the general fungal taxonomy. Our Dothideomycetes data included four orders: Dothideales, Pleosporales, Capnodiales, and Botryosphaeriales, and none of the orders were monophyletic in the both phylogenetic trees. Although we used Mucoromycotina as a outgroup taxa in our trees, *Ustilago maydis* clustered with the Mucoromycotina clade.

#### Combined tree

**Figure 6:**
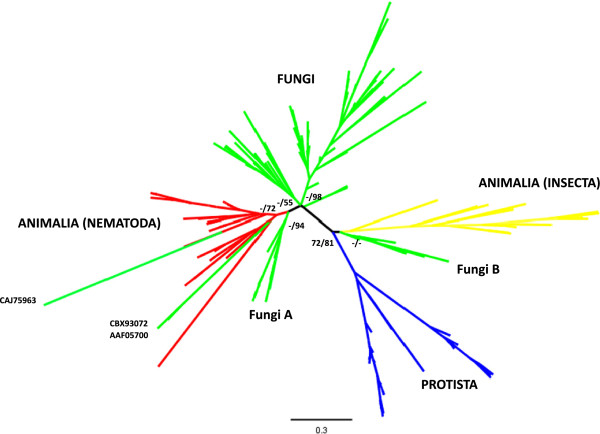
**Best tree of Maximum Likelihood analysis using the program RAxML-VI-HPC v. 4.0.0. with GH45 sequences.** The amino acid sequences from Animalia, Fungi and Protista Kingdoms were used, and LG + G model was conducted in the analysis. Bootstrap values and posterior probabilities are showed on supported major clades.

A broad phylogenetic tree was generated using the GH45 sequences from nematodes and fungi obtained in this study and other sequences from CAZy database (http://www.cazy.org/). Sequences from bacteria and molluscs were not included as they are very different compared to sequences from other organisms (Figure 6). The ML best tree showed a monophyletic clade for nematodes and also for protists and insects, while the fungi sequences are distributed into several separated clades. However, the clades formed are weakly supported with the exception of prostists and insects. Deep clades are usually well supported in our analysis. Some sequences from fungi were located in the Nematoda clade (CAJ75963 [*Rasamsonia emersonii*]; CBX93072 [*Leptosphaeria maculans*]; AAF05700 [*Alternaria alternate*]). A fungal clade which is most closely related to the nematode sequences and on the basal position of the nematode clade in the tree (labeled as FungiA in Figure 6) consisted of sequences from Sordariomycetes fungi (Figure 5 and Additional file 2: Figure S3). Another fungal clade positioned next to insect clade (labeled as FungiB in Figure 6) was made up of fungi belonging to Mucoromycotina and Basidiomycetes.

## Discussion

In this study we have identified 44 and 70 novel GH45-like sequences from nematodes and fungi respectively, as a result of a wide range screening programme. This is equivalent to two thirds of the eukaryotic GH45 genes previously described (CAZy: http://www.cazy.org/). Most of these sequences are from species for which there was no prior knowledge of GH45 cellulases. This study has therefore greatly increased the available information about distribution of GH45 sequences in eukaryotes.

Our study suggests distribution of GH45 genes in nematodes is likely to be restricted in a single phylogenetic group that includes the families Parasitaphelenchidae and Aphelenchoididae as the sequences have been found only from *Bursaphelenchus* species, *Ruehmaphelenchus* sp. and *A. besseyi*. No GH45-like sequence was detected from other nematode species including *Pratylenchus* sp. and *Aphelenchus* sp. by PCR amplification in this study and no sequence showing similarity to GH45 genes has been identified in the extensive genome, EST or RNA-seq sequences from any other nematodes including *C. elegans, Meloidogyne* species and *A. avenae.* However, there are still many nematode genera, including plant-parasitic species, which have not yet been subjected to detailed analysis. The possibility that other species which have not yet been analysed also have GH45-like sequences therefore remains.

In fungi, GH45 sequences were found from a variety of Ascomycota species, ranging from Sordariomycetes to Saccharomycetes. Only a small number of GH45 sequences have previously been reported from Basidiomycetous fungi and no PCR amplification of these sequences was observed from any of the Basidiomycetous fungi used in this study, suggesting GH45 genes were more widely distributed in Ascomycota than in Basidiomycota. The phylogenetic trees of fungal GH45 sequences showed good consistency with the LSU tree in terms of species relationships, although some duplications in GH45 sequences were observed in some specific clades and they show a nested structure in the tree. Therefore it seems likely the GH45 genes were inherited vertically and evolved from a common ancestor of these fungi. Currently seven phyla in the kingdom Fungi are proposed: Microsporidia, Chrytridiomycota, Blastocladiomycota, Neocallimastigomycota, Glomeromycota, Ascomycota, and Basidiomycota [19]. In this study we used large numbers of species mainly from two big fungal groups (Ascomycota and Basidiomycota). It would be interesting to investigate distributions of GH45 genes in other phyla as they still remain unclear.

The phylogenetic trees of nematode GH45 sequences showed a more complex structure than those of the fungi. The fact that several different copies of the genes are present within each individual nematode makes it difficult to interpret the phylogenetic trees. For example, we found 4 distinct GH45 sequences in *B. luxuriosae* and *B. purvispicularis* and in each case subsets of these sequences are nested with sequences from other species within the trees (Figure 3). In addition we observe several small clusters comprised by multiple sequences from one species within the tree (Figure 3). These patterns are consistent with an initial expansion of the gene family in the common ancestor of the *Bursaphelenchus* genus followed by further expansions within individual clades and species.

The genome sequence of *B. xylophilus* revealed the species has 11 GH45 genes in the genome [14] and in our tree they were separated into 5 clusters (Figure 3). This adds further weight to the suggestion that expansions have occurred both before and after speciation. Some of those genes have another intron position (p8) that was not found in other nematode species using PCR screening, suggesting p8 genes in other species might be missed in the PCR process possibly because of primer mismatches. A more complete set of cellulase genes of these nematode species will enable a more detailed analysis of the evolution of these genes in nematodes.

Despite the complex structures of the phylogenetic trees generated there is some correlation between the phylogeny based on rDNA SSU sequences and the GH45 trees. A phylogenetic clade in the GH45 trees which includes the *xylophilus* group and a clade with *B. kiyoharai* and *Bursaphelenchus* sp2 are both well supported and these clades are also well supported in the tree obtained using SSU rDNA. This again suggests the distribution of GH45 cellulases in *Bursaphelenchus* species is originated from a common ancestor followed by expansions of the genes during the evolution.

We found two interesting inconsistencies between the GH45 tree and the SSU tree. GH45 sequences of *B. parvispicularis* are clustered together with those from the *xylophilus* group while the SSU of the species is phylogenetically closer to bark beetle/weevil associates (*B. yongensis*, *B. poligraphi*, *Bursaphelenchus* sp2 and sp3.). Although we do not have a clear explanation for this inconsistency, this might be related with their biological characters, e.g., carrier insect and habitat environments. The species in the *xylophilus* group are associated with cerambycid beetles and inhabit relatively deep wood (in humid conditions), and the other smaller insect associates inhabit shallow wood (in dry conditions). Although the detailed life history of *B. parvispicularis* has not been examined, the habitat preference might be closer to that of the *xylophilus* group. The detailed biological analysis of *B. parvispicularis* may give an insight into the function of these genes in relation to nematodes’ biological characters.

The GH45 sequence from *A. besseyi* is in a closer position to the sequences from the *Bursaphelenchus* species than those from *Ruehmaphelenchus* sp. in the tree. As seen in the SSU tree, *A. besseyi* is thought to be more distantly related to *Bursaphelenchus* species than other *Aphelenchus* and *Ruehmaphelenchus* species (Figure 2). *Aphelenchoides* species are mainly fungal-feeders and are thought to have less association with plants than *Bursaphelenchus.* However, *A. besseyi* is one of a few species which are known to be parasitic to plants. It would be interesting to examine GH45 genes in other *Aphelenchoides* species to have insights into the evolution of parasitism in this nematode genus.

GH45 sequences are widely represented in other organisms including bacteria, protists, insects and molluscs [20]. Sequences from molluscs and bacteria were excluded from this analysis because of their low sequence similarities to GH45 sequences from other species. The genes from these groups are thought to comprise a subfamily within the GH45 family. Our broad phylogeny of GH45 sequences showed a clear grouping of organisms in the best tree obtained (Figure 6). Sequences from insects and protists presented monophyletic clades in the tree. Taking into account of the position of nematodes in the tree and the presence of the genes in *Ruehmaphelenchus* sp. and *A. besseyi* it is possible that the HGT event that gave rise to GH45 sequences in nematodes occurred in an early ancestral species in Aphelenchoidea from a close contact with Ascomycota fungi species as previously suggested [9,14]. We hypothesise that the nematodes acquired GH45 genes from one of Sordariomycetous fungi as sequences from these fungi are located at the basal position of the all nematode sequences in the tree (Figure 6). The majority of the Aphelenchoidea species are fungal-feeders and some of the species live in a densely fungal populated environment.

The GH45 sequences from fungal species (CAJ75963 [*Rasamsonia emersonii*]; CBX93072 [*Leptosphaeria maculans*]; AAF05700 [*Alternaria alternata*]) are clustered with the sequences from the Nematoda. This is difficult to explain and may have arisen due to an artifact or, more speculatively, due to sequence changes reflecting functional restrictions on the cellulases within these species.

The nematode GH45 sequences described here have three types of intron positions (p0, p8 and p11). Intriguingly position 11 is shared by the nematodes and some fungi. Although introns have high mutation rates, making it difficult to trace lineages through sequence similarity, their positions are well conserved and can provide strong evidence to support conclusions on the origins of the genes [21]. Therefore the fact that nematode and fungal genes share an intron position in some cases suggests a common origin of these genes. Indeed most of the fungal species (ten out of 15) that possess this intron position belong to Letiomycetes and cluster into one clade in the tree (Figure 4). In addition, the aforementioned sequences from Sordariomycetes fungi (labeled FungiA in Figures 5 and 6 and Additional file 2: Figure S3) also have a position 11 intron as well as genes with no intron. This can be a support for the hypothesis that the source of the nematode genes is likely to be a Sordariomycetes fungus.

Another family of cellulases is represented in other groups of plant-parasitic nematodes. All members of clade 12 of phylum Nematoda analysed to date harbor one or multiple GH5 cellulases [22]. In the Tylenchid plant parasites this family of GH5 cellulases is thought to have been derived from bacteria [22] while in the case of *Pristonchus* spp. (Clade 9, Diplogasteridae) the GH5 sequences present are more likely to have been acquired from an amoebozoan or related microorganism [23]. The presence of GH5 has also been identified in the fungivorous nematode *A. avenae*[24]. The phylogenetic position of this species was controversial until recently but now it is clearly accepted that *Aphelenchus* is more closely related to Tylenchida than to *Aphelenchoides* and *Bursaphelenchus* based on the comprehensive molecular phylogenetic study by Van Megen et al. [8]. Our tree also supports this phylogenetic position (Figure 2).

*Bursaphelenchus* species are likely to have GH45 cellulases regardless of their pathogenicity to plants. The only species proven to be pathogenic in natural conditions from our dataset of studied sequences is *B. xylophilus*[14], with only a few other species demonstrated as weak disease agents under certain environmental conditions [25]. Most other species, including *Ruehmaphelenchus,* are associated only with dead trees. Our finding of the widespread occurrence of GH45 cellulases across the *Bursaphelenchus* genus and in a closely related genus (*Ruehmaphelenchus*) suggests GH45 cellulases are used by nematodes to soften the cell walls of plants regardless of whether the nematode is a pathogen or simply a fungal feeder that moves through dead plants to locate food.

## Conclusions

It used to be believed that animals (Metazoa) do not have endogenous cellulase (endo-beta-1,4-glucanase) and rely on their symbiotic microorganisms for cellulose digestion. However, it is now clear that some invertebrate species, including nematodes, have endogenous cellulase genes which produce enzymes to digest cellulose.

In order to investigate distribution and evolution of GH45 cellulase genes in nematodes and fungi we performed a wide ranging screen and intensive phylogenetic analysis of GH45 sequences. We identified 44 novel sequences from a small group of nematode species and 77 from a wide variety of Ascomycetous fungi, indicating a wide distribution of GH45 cellulases in Ascomycetous fungi and so far been found in a single major nematode lineage. The close relationships between the sequences from nematodes and Ascomycetous fungi, as well as the conserved gene structures gave us the reasonable hypothesis that nematode GH45 cellulase genes were acquired via HGT from fungi probably belonging to class Sordariomycetes.

## Methods

### Biological materials

The fungal strains and nematode species used in this study were from the culture collection stored at the Forest Pathology Lab in FFPRI or from NIAS Genebank culture collection (Additional file 1: Table S1, S2).

### DNA extraction

Fungi were cultured on cellophane membranes placed on potato dextrose agar (PDA, Eiken Chemical) plates at 23°C for periods appropriate for each fungus. Fungal mycelium was harvested from each plate by scratching the surface of the membranes using small metal spatulas. The mycelium was either immediately used for DNA extraction or stored at −80°C for further use. Genomic DNAs from fungi were extracted using a rapid and high-throughput extraction method [26].

Nematodes were cultured at 25°C on *Botrytis cinerea* grown on potato dextrose agar plates. Nematodes were collected using a modified Baermann funnel technique [27] and cleaned in several rinses of 0.5x PBST before use. Genomic DNAs were extracted as described in Kikuchi et al. [26].

### PCR amplification and Sequencing

Two degenerate primers, GHF45-1f and GHF45-2r were designed from a highly conserved region of GHF45 cellulases selected from CAZy homepage (Carbohydrated Active EnZYmes; http://www.cazy.org/). The sequence of GHF45-1f is based on TRYWDCC (amino acids 22–28 in unprocessed *B. xylophilus* Bx-ENG1). The sequence of GHF45-2r is based on PGGG(F/V)GA (amino acids 141–147 in unprocessed *B. xylophilus* Bx-ENG1) (Additional file 2: Figure S1).

Amplification was performed using GoTaq green master mix (Promega) or IQ SYBR Green Supermix (BioRad) with 0.5 μM of each primer and appropriately diluted genomic DNA solution. After checking the bands on a 1% agarose gel, PCR products were cloned into pGEM-Teasy vector (Promega) and transformed into *E. coli* (DH5alpha). Sixteen *E. coli* clones from each product were picked randomly from the plate and sequenced from both ends using BigDye Terminator ver 3.1 (Life Technologies).

The small subunit of ribosomal RNA genes of nematodes were amplified using primers F07 (5′-AAAGATTAAGCCATGCATG-3′) and nR (5′-TTACGACTTTTGCCCGGTTC-3′). Large subunit of ribosomal RNA genes of fungi were amplified with primer LR0R and LR5 (a location map and oligonucleotide sequences of these primers can be found at http://www.biology.duke.edu/fungi/mycolab/primers.htm). Amplified products were cleaned with Minelute 96 plate (Qiagen) and sequenced from the both ends.

### Intron prediction and RT-PCR

Intron prediction was performed using SpliceView (http://www.itb.cnr.it/webgene/) with an option of organism=“*Caenorhabditis elegans*” or organism=“*Aspergillus niger*” for nematodes and fungi respectively and manually adjusted on the basis of conserved regions of GH45 cellulase sequences.

RNA was extracted from nematodes which were cultured on *B. cinerea* as described in Kikuchi et al. [28] and cDNA was synthesized using iScript (BioRad) following the manufacturer’s instructions. The GH45 cellulase cDNA was PCR amplified using primers designed for the genomic DNA fragment and sequenced to confirm the exon/intron structures of the fragments.

### Phylogenetic analyses

GH45 cellulase sequences were obtained from the CAZy homepage (http://www.cazy.org) and GenBank database in addition to the sequences obtained in this study. Specific phylogenies of nucleotides and amino acids from GH45 coding sequences were studied in the *Bursaphelenchus* genus using *Ruehmaphelenchus* sp. as an outgroup, while for the fungi phylogeny *Phycomyces nitens* was used as an outgroup. A combined phylogenic tree was also constructed using 208 GH45 amino acid sequences (including all sequences obtained in this study). Bacteria and mollusc sequences were excluded from the analysis. Phylogenetic trees based on ribosomal sequences from fungi and nematodes were made using sequences obtained in this study and sequences from GenBank. Nucleotide and amino acid sequences for the different phylogenetic analysis were aligned using ClustalX2 [29] and MUSCLE [30], respectively. The best fitting model of protein and DNA evolution were obtained based on the AIC (Akaike Information Criterion) using ProtTest 2.4 server [31] and jModelTest v. 2 [32], respectively. Models for the different alignments were as follows: (i) Nematode ribosomal sequences: GTR + I + G; (ii) Nematode GH45 DNA sequences: 010234 + I + G + F; (iii) Nematode GH45 aminoacid sequences: WAG + I + G + F; (iv) Fungi ribosomal sequences: TIM3 + G; (v) Fungi GH45 DNA sequences: TVM + I + G; (vi) Fungi GH45 aminoacid sequences: LG + I + G. Phylogenetic analysis of the sequence data sets were performed with maximum likelihood (ML) using the program RAxML-VI-HPC v. 4.0.0 (Randomized Accelerated Maximum Likelihood for High Performance Computing) [33] using 500 bootstraps and the computed model. Bayesian inference (BI) was conducting using MrBayes 3.1.2 [34] using aforementioned site-specific models. Four chains were run for a minimum of 4 × 10^6^ generations and two independent runs were performed. After discarding burn-in samples and evaluating convergence, the remaining topologies were used to generate a 50% majority-rule consensus tree. Trees were visualised using FigTree v1.4.0 (http://tree.bio.ed.ac.uk/software/figtree/).

## Availability of supporting data

Sequences obtained in this study have been deposited to Genbank under accession nos: KF590043-KF590214.

## Abbreviations

: Bursaphelenchus sp. 3 is now described as Bursaphelenchus niphades [15]; GH: Glycoside hydrolase; HGT: Horizontal gene transfer.

## Competing interests

The authors declare that they have no competing interests.

## Authors’ contributions

JEP and YH carried out the phylogenetic analyses, participated in drafting the manuscript. JIT and AH carried out gene structure analysis and drafted the manuscript. HM and NH participated in the phylogenetic analyses. JTJ and TK conceived of the study, participated in its design and coordination and drafted the manuscript. All authors read and approved the final manuscript.

## Supplementary Material

Additional file 1: Table S1 Nematode cultures used in this study. **Table S2.** Fungi cultures used in this study. **Table S3.** Nematode GH45 sequences obtained in this study. **Table S4.** Fungal GH45 sequences obtained in this study. **Table S5.** Sequences and Genbank accession numbers used in Figure 4. **Table S6.** Species names and GenBank accession numbers used in Figure 6. Click here for file

Additional file 2: Figure S1 Amino acid alignment of GH45 proteins and intron positions. Intron positions are indicated by triangles on the alignment. Conserved regions used to design primers are boxed. Asterisks indicate the two catalytic core residues (Asp, Asp). The numbers to the left indicate the amino acid position of the respective proteins. Phase of the introns is shown by distinct triangles. BxENG1, 2 and 3 – *B. xylophilus* sequences (nematode – BAD34543-5), B_ciner1 – GHF45 cellulase from *Botrytis cinerea* (fungus – CCD33730), R.oryza1 – *Rhizopus oryzae* (fungus – BAC53956), H_insol1 – *Humicola insolens* (fungus – CAB42307), A.germ1 – *Apriona germari* (insect – AAN78326). **Figure S2.** Bayesian 50% majority rule consensus tree of GH45 amino acid sequences from Aphelenchoidea under WAG + I + G + F model. Posterior probabilities more than 65% are given for appropriate clades; bootstrap values greater than 50% are given on appropriate clades in ML analysis. **Figure S3.** Bayesian 50% majority rule consensus tree of GH45 amino acid sequences from Ascomycota, Basidiomycota and Zygomycota under LG + I + G model. Posterior probabilities more than 65% are given for appropriate clades; bootstrap values greater than 50% are given on appropriate clades in ML analysis. Click here for file
