# Supplementary material for: Distribution and evolution of glycoside hydrolase family 45 cellulases in nematodes and fungi
Source: BMC Evol Biol. 2014 Apr 1;14:69. doi: 10.1186/1471-2148-14-69 (PMC3997829; doi:10.1186/1471-2148-14-69)
Supplement: Additional file 1: Table S1 — Nematode cultures used in this study. Table S2. Fungi cultures used in this study. Table S3. Nematode GH45 sequences obtained in this study. Table S4. Fungal GH45 sequences obtained in this study. Table S5. Sequences and Genbank accession numbers used in Figure 4. Table S6. Species names and GenBank accession numbers used in Figure 6. [file 1471-2148-14-69-S1.pdf]

Table S1. Nematode cultures used in this study

DNA_ID	Genus	Species	Strain/Populati	Source
Ka4C1	*Bursaphelenchus*	*xylophilus*	Ka4C1	*Pinus thunbergii*
AaF1	*Aphelenchus*	*avenae*	AaF1	-
Pra	*Pratylenchus*	sp.	okd-x	-
201	*Aphelenchoides*	sp.	NK201	*Fagus crenata*
202	*Ruehmaphelenchus*	sp.	NK202	*Xyleborus ganshoensis*
203	*Bursaphelenchus*	sp. 3	NK203	*Niphades variegatus*
204	*Bursaphelenchus*	*doui*	NK204	*Monochamus subfasciatus*
205	*Bursaphelenchus*	*luxuriosiae*	NK205	*Acalolepta luxuriosa*
206	*Bursaphelenchus*	sp. 1	NK206	*Pinus kesia*
207	*Bursaphelenchus*	sp. 3	NK207	*Niphades variegatus*
208	*Bursaphelenchus*	*xylophilus*	NK208	*Monochamus alternatus*
209	*Bursaphelenchus*	*yongensis*	NK209	*Pinus thunbergii*
210	*Bursaphelenchus*	sp. 2	NK210	*Cryphalus sp.*
212	*Bursaphelenchus*	*okinawaensis*	NK212	*Monochamus maruokai*
215	*Bursaphelenchus*	*kiyoharai*	NK215	*Xyleborus seriatus*
216	*Bursaphelenchus*	*purviscularis*	NK216	*Quercus crispula*
217	*Bursaphelenchus*	*doui*	NK217	*Acalolepta fraudatrix*
220	*Bursaphelenchus*	sp. 3	NK220	*Abies veitchii*
221	*Bursaphelenchus*	*kiyoharai*	NK221	*Fagus crenata*
222	*Aphelenchoides*	*xylocopae*	NK222	*Xylocopa sp.*
223	*Aphelenchoides*	sp.	NK223	*Xylocopa sp.*
224	*Bursaphelenchus*	*purviscularis*	NK224	*Picea koyamae*
225	*Bursaphelenchus*	*poligraphi*	NK225	*Pinus thunbergii*
Bc	*Bursaphelenchus*	*conicaudatus*	NKBc	-
Bl	*Bursaphelenchus*	*luxuriosiae*	Nara	*Acalolepta luxuriosa*
Para	*Parasitaphelenchus*	sp.	NKPara	-

Table S2. Fungi cultures used in this study

DNA_ID	Genus	Species	Strain ID (FFGC ID)	Other IDs	Phylum
FFGC07_01A01	*Alternaria*	*longipes*	A 1-7		Ascomycota
FFGC07_01B01	*Alternaria*	*tenuis*	A 1-9		Ascomycota
FFGC07_01C01	*Actinopelte*	*dryina*	A 3-2		Ascomycota
FFGC07_01D01	*Ascochyta*	sp.	A 4-1		Ascomycota
FFGC07_01E01	*Ascochyta*	*pisi*	A 4-4		Ascomycota
FFGC07_01F01	*Arthrinium*	*phaeospermum*	A 5-1		Ascomycota
FFGC07_01G01	*Amylostereum*	*areolatum*	A 9-2		Basidiomycota
FFGC07_01H01	*Armillariella*	*melea*	96-41		Basidiomycota
FFGC07_01A02	*Armillariella*	*tabescens*	96-1-8		Basidiomycota
FFGC07_01B02	*Armillariella*	*ostoyae*	94-8-12		Basidiomycota
FFGC07_01C02	*Armillariella*	*tabescens*	94-7		Basidiomycota
FFGC07_01D02	*Botryosphaeria*	*dothidea*	B 1-15		Ascomycota
FFGC07_01E02	*Botryosphaeria*	sp.	B 1-16		Ascomycota
FFGC07_01F02	*Xenostigmina*	sp.	B 4-1		Ascomycota
FFGC07_01G02	*Bactridium*	sp.	B 6-1		Ascomycota
FFGC07_01H02	*Botrytis*	*cinerea*	BB-6		Ascomycota
FFGC07_01A03	*Cytospora*	*abietis*	C 1-6		Ascomycota
FFGC07_01B03	*Cytospora*	*kunzei*	C 1-15		Ascomycota
FFGC07_01C03	*Colletotrichum*	*gloeosporioides*	C 2-26		Ascomycota
FFGC07_01D03	*Colletotrichum*	*truncatum*	C 2-72		Ascomycota
FFGC07_01E03	*Cryptodiaporthe*	*raveneliana*	C 3-1		Ascomycota
FFGC07_01F03	*Cryptodiaporthe*	*castanea*	C 3-8		Ascomycota
FFGC07_01G03	*Cylindrocladium*	*scoparium*	C 4-1		Ascomycota
FFGC07_01H03	*Calonectria*	*kyotensis*	C 4-8		Ascomycota
FFGC07_01A04	*Coniothyrium*	sp.	C 5-14		Ascomycota
FFGC07_01B04	*Coniothyrium*	*concentricum*	C 5-15		Ascomycota
FFGC07_01C04	*Corticium*	*rolfsii*	C 6-6		Basidiomycota
FFGC07_01D04	*Cenangium*	sp.	C 7-1		Ascomycota
FFGC07_01E04	*Davidiella_*	sp.	C 7-7		Ascomycota
FFGC07_01F04	*Curvularia*	*pallescens*	C 8-2		Ascomycota
FFGC07_01G04	*Curvularia*	*lunata*	C 8-3		Ascomycota
FFGC07_01H04	*Coryneum*	*modonium*	C 9-2		Ascomycota
FFGC07_01A05	*Coryneum*	sp.	C 9-3		Ascomycota
FFGC07_01B05	*Cladosporium*	sp.	C 11-3		Ascomycota
FFGC07_01C05	*Corynespora*	*cassiicola*	C 13-1		Ascomycota
FFGC07_01D05	*Cylindrosporium*	sp.	C 14-3		Ascomycota
FFGC07_01E05	*Cercosporella*	*juglandis*	C15-1		Ascomycota
FFGC07_01F05	*Cercosporella*	sp.	C 15-6		Ascomycota
FFGC07_01G05	*Camasosporium*	*mori*	C 18-2		Ascomycota
FFGC07_01H05	*Camasosporium*	sp.	C 18-3		Ascomycota
FFGC07_01A06	*Ceratocystis*	*ips*	C 25-1		Ascomycota
FFGC07_01B06	*Chlorosrypha*	*chamaecyparidis*	C 26-1		Ascomycota
FFGC07_01C06	*Crypotosporiopsis*	sp.	C 27-16		Ascomycota
FFGC07_01D06	*Crypotosporiopsis*	*abietina*	C 27-21		Ascomycota
FFGC07_01E06	*Cylindrocarpon*	sp.	C 28-19		Ascomycota
FFGC07_01F06	*Cylindrocarpon*	*destructans*	C 28-21		Ascomycota
FFGC07_01G06	*Cryptocline*	sp.	C 29-2		Ascomycota
FFGC07_01H06	*Coniella*	*castaneicola*	C 30-1		Ascomycota
FFGC07_01A07	*Coniocaeta*	sp.	C 31-1		Ascomycota
FFGC07_01B07	*Cercospora*	*populicola*	CB-2		Ascomycota
FFGC07_01C07	*Diplodia*	sp.	D 1-5		Ascomycota
FFGC07_01D07	*Diplodia*	*pinea*	D 1-7		Ascomycota
FFGC07_01E07	*Diplodia*	*pinea*	D 1-14		Ascomycota
FFGC07_01F07	*Diatrypella*	sp.	D 2-1		Ascomycota
FFGC07_01G07	*Diatrype*	sp.	D 3-8		Ascomycota
FFGC07_01H07	*Diplodina*	*populi*	D 4-1		Ascomycota
FFGC07_01A08	*Dothidea*	sp.	D 5-2		Ascomycota
FFGC07_01B08	*Dothidea*	*sambuci*	D 5-3		Ascomycota
FFGC07_01C08	*Didymella*	sp.	D 6-3		Ascomycota
FFGC07_01D08	*Diaporthopsis*	sp.	D 8-2		Ascomycota
FFGC07_01E08	*Dothiorella*	sp.	D 11-5		Ascomycota
FFGC07_01F08	*Leptosphaerulina*	sp.	D 12-1		Ascomycota
FFGC07_01G08	*Diaporthe*	*medusaea*	DB-17		Ascomycota
FFGC07_01H08	*Cryphonectria*	*parasitica*	EP-1		Ascomycota
FFGC07_01A09	*Cryphonectria*	*radicalis*	E-8		Ascomycota
FFGC07_01B09	*Endothiella*	sp.	E 1-3		Ascomycota
FFGC07_01C09	*Epicoccum*	sp.	E 2-5		Ascomycota
FFGC07_01D09	*Epicoccum*	*nigrum*	E 2-14		Ascomycota
FFGC07_01E09	*Entomosporium*	*mespili*	E 4-2		Ascomycota
FFGC07_01F09	*Entomosporium*	sp.	E 4-4		Ascomycota
FFGC07_01G09	*Exobasidium*	*pentasporium*	E 5-3		Basidiomycota
FFGC07_01H09	*Exobasidium*	*bisporum*	E 5-4		Basidiomycota
FFGC07_01A10	*Fusarium*	*oxysporum*	FB-11		Ascomycota

FFGC07_01D10	*Fusicoccum*	*castaneum*	F 1-1	Ascomycota
FFGC07_01E10	*Fusicoccum*	sp.	F 1-5	Ascomycota
FFGC07_01F10	*Guignardia*	*laricina*	GC-74	Ascomycota
FFGC07_01G10	*Gloeosporium*	*kawakamii*	G 1-27	Ascomycota
FFGC07_01H10	*Gloeosporium*	sp.	G 1-28	Ascomycota
FFGC07_01A11	*Glomerella*	*cingulata*	G 2-1	Ascomycota
FFGC07_01B11	*Glomerella*	*ochraeum*	G 2-21	Ascomycota
FFGC07_01C11	*Gbiocladium*	sp.	G 3-1	Ascomycota
FFGC07_01D11	*Gnomonia*	*setacea*	G 4-1	Ascomycota
FFGC07_01E11	*Pyrenochaeta*	sp.	H 1-3	Ascomycota
FFGC07_01F11	*Helminthosporium*	*oryzae*	H 2-1	Ascomycota
FFGC07_01G11	*Helminthosporium*	sp.	H 2-3	Ascomycota
FFGC07_01H11	*Helicobasidium*	*purpureum*	H-4	Basidiomycota
FFGC07_01A12	*Hendersonia*	sp.	H 4-3	Ascomycota
FFGC07_01B12	*Heterosporium*	*phlei*	H 6-1	Ascomycota
FFGC07_01C12	*Hulbania*	*juniperi*	H 9-1	Ascomycota
FFGC07_01D12	*Helicobasidium*	*mompa*	H-12	Basidiomycota
FFGC07_01E12	*Lophodermium*	*pinastri*	L-3	Ascomycota
FFGC07_01F12	*Pestalotiopsis*	sp.	L 4-1	Ascomycota
FFGC07_01G12	*Lachnellula*	sp.	L 5-2	Ascomycota
FFGC07_01H12	*Leptographium*	*truncatum*	L 6-1	Ascomycota
FFGC07_02A01	*Leptographium*	*procerum*	L 6-2	Ascomycota
FFGC07_02B01	*Ophiostoma*	*minus*	MCC-TA	Ascomycota
FFGC07_02C01	*Leptographium*	*wingfieldii*	MCC-TB	Ascomycota
FFGC07_02D01	*Macrophoma*	*sugi*	M 1-1	Ascomycota
FFGC07_02E01	*Macrophoma*	*quercicola*	M 1-9	Ascomycota
FFGC07_02F01	*Myxosporium*	*rhois*	M 2-2	Ascomycota
FFGC07_02G01	*Myxosporium*	sp.	M 2-13	Ascomycota
FFGC07_02H01	*Melanconium*	*oblongum*	M 3-1	Ascomycota
FFGC07_02A02	*Melanconis*	*juglandis*	M 4-1	Ascomycota
FFGC07_02B02	*Melanconis*	*stilbostoma*	M 4-2	Ascomycota
FFGC07_02C02	*Monochaetia*	*monochaeta*	M 5-2	Ascomycota
FFGC07_02D02	*Seiridium*	*unicorne*	M 5-7	Ascomycota
FFGC07_02E02	*Macrosporium*	sp.	M 7-1	Ascomycota
FFGC07_02F02	*Metasphaeria*	sp.	M 11-1	Ascomycota
FFGC07_02G02	*Monilia*	*kusanoi*	M 13-1	Ascomycota
FFGC07_02H02	*Monostichella*	sp.	M 15-2	Ascomycota
FFGC07_02A03	*Melanospora*	sp.	M 16-1	Ascomycota
FFGC07_02B03	*Macrophomina*	*phaseolina*	M 17-1	Ascomycota
FFGC07_02C03	*Mycosphaerella*	*togashiana*	MB-1	Ascomycota
FFGC07_02D03	*Mycosphaerella*	*myricae*	MB-13	Ascomycota
FFGC07_02E03	*Mycosphaerella*	*myricae*	MB-14	Ascomycota
FFGC07_02F03	*Mycosphaerella*	*luzonensis*	MB-15	Ascomycota
FFGC07_02G03	*Mundkurella*	*kalopanacis*	MU-1	Basidiomycota
FFGC07_02H03	*Mycosphaerella*	*sojae*	MA-1	Ascomycota
FFGC07_02A04	*Nectria*	*cinnabarina*	N 20	Ascomycota
FFGC07_02B04	*Nectria*	sp.	N 24	Ascomycota
FFGC07_02C04	*Neocosmospora*	sp.	N 1-4	Ascomycota
FFGC07_02D04	*Ophiostoma*	*piceae*	OP-1	Ascomycota
FFGC07_02E04	*Ophiovalsa*	*femoralis*	O 1-1	Ascomycota
FFGC07_02F04	*Ophiostoma*	sp.	OP-4	Ascomycota
FFGC07_02G04	*Pestalotiopsis*	*guepini*	P1B-1	Ascomycota
FFGC07_02H04	*Pestalotia*	*populi-nigrae*	P1B-8	Ascomycota
FFGC07_02A05	*Pestalotia*	*aceris*	P1B-29	Ascomycota
FFGC07_02B05	*Phomopsis*	*rudis*	PB-6	Ascomycota
FFGC07_02C05	*Epicoccum*	sp.	P 2-20	Ascomycota
FFGC07_02D05	*Phyllosticta*	*alcides*	P 3-4	Ascomycota
FFGC07_02E05	*Patellina*	sp.	P 10-1	Ascomycota
FFGC07_02F05	*Pestalotiopsis*	*foedans*	P 18-1	Ascomycota
FFGC07_02G05	*Phialophora*	sp.	P 23-1	Ascomycota
FFGC07_02H05	*Pestalotiopsis*	*glandicola*	P1C-9	Ascomycota
FFGC07_02A06	*Phaeoseptoria*	*eucalypti*	P 24-1	Ascomycota
FFGC07_02B06	*Rhizosphaera*	sp.	R 2-13	Ascomycota
FFGC07_02C06	*Melanomma*	sp.	R 5-1	Ascomycota
FFGC07_02D06	*Rhizina*	*undulata*	R 6-1	Ascomycota
FFGC07_02E06	*Retinocyclus*	sp.	R 7-12	Ascomycota
FFGC07_02F06	*Sarea*	*resinae*	R 7-23	Ascomycota
FFGC07_02G06	*Thanatephorus*	*cucumeris*	RC-4	Basidiomycota
FFGC07_02H06	*Rhizosphaera*	*kalkhoffii*	R 2-2	Ascomycota
FFGC07_02A07	*Septotinia*	*populiperda*	S 2-4	Ascomycota
FFGC07_02B07	*Septotis*	*populiperda*	S 2-3	Ascomycota
FFGC07_02C07	*Septotis*	sp.	S 2-9	Ascomycota
FFGC07_02D07	*Sclerotinia*	*sclerotiorum*	S 4-5	Ascomycota
FFGC07_02E07	*Sclerotinia*	*camelliae*	S 4-11	Ascomycota
FFGC07_02F07	*Septoria*	*pini-thunbergii*	S 5-20	Ascomycota

FFGC07_02A08	Stagonospora	sp.	S 8-4		Ascomycota
FFGC07_02B08	Stagonospora	cinnamomum	S 8-7		Ascomycota
FFGC07_02C08	Selenophoma	sp.	S 12-3		Ascomycota
FFGC07_02D08	Strasseriopsis	tsugae	S 13-1		Ascomycota
FFGC07_02E08	Scolicosporium	sp.	S 16-1		Ascomycota
FFGC07_02F08	Tubercularia	vulgaris	T 1-5		Ascomycota
FFGC07_02G08	Trichothecium	roseum	T 2-2		Ascomycota
FFGC07_02H08	Tuberculis	sp.	T 6-1		Ascomycota
FFGC07_02A09	Tubakia	dryina	T 7-1		Ascomycota
FFGC07_02B09	Trochophora	simplex	T 9-1		Ascomycota
FFGC07_02C09	Truncatella	sp.	T 10-3		Ascomycota
FFGC07_02D09	Trichoscyphella	resinaria	T-23		Ascomycota
FFGC07_02E09	Valsaria	sp.	V 2-1		Ascomycota
FFGC07_02F09	Verticillium	sp.	V 3-1		Ascomycota
FFGC07_02G09	Pyrenochaeta	cava	V-44		Ascomycota
FFGC07_02H09	Leucostoma	niveum	V-51		Ascomycota
FFGC07_02A10	Leucostoma	persoonii	V-66		Ascomycota
FFGC07_02B10	Valsa	paulowniae	V-77		Ascomycota
FFGC07_02C10	Paraphoma	sp.	Z-1		Ascomycota
FFGC07_02D10	Eupenicillium	javanicum	FRR 2859		Ascomycota
FFGC07_02E10	Helotium	leucellum	C 24-1		Basidiomycota
FFGC07_02F10	Penicillium	megasporum	P 2259		Ascomycota
FFGC07_02G10	Penicillium	pseudostrouaticum	P 2249		Ascomycota
FFGC07_02H10	Penicillium	verrucosum	P2263		Ascomycota
FFGC07_02A11	Rhizoctonia	sp.	RZ-01		Basidiomycota
FFGC07_02B11	Rhizoctonia	sp.	RZ-02		Basidiomycota
FFGC07_02C11	Chaetomium	sp.	Ch-1		Ascomycota
FFGC07_02D11	Penicillium	citrinum	T 16-2		Ascomycota
FFGC07_02E11	Trochophora	simplex	H 8-1		Ascomycota
FFGC07_02F11	Guignardia	alnigena	GB-16		Ascomycota
FFGC07_02G11	Cercospora	sequoiae	CC-85		Ascomycota
FFGC07_02H11	Marssonina	brunnea	M 14-1		Ascomycota
FFGC07_02A12	Bartalinia	robillardoides	B 5-1		Ascomycota
FFGC07_02B12	Taphrina	wiesneri	T 8-1		Ascomycota
FFGC07_02C12	Taphrina	wiesneri	T8-0173		Ascomycota
FFGC07_02D12	Amylostereum	areolatum	A 9-1		Basidiomycota
FFGC07_02E12	Ascocalyx	pinicola	A 8-2		Ascomycota
FFGC07_02F12	Lachnellula	calyciformis	L 5-5		Ascomycota
FFGC07_02G12	Plectosphaera	cryptomeriae	P 17-1		Ascomycota
FFGC07_02H12	Hysterium	sp.	H 10-1		Ascomycota
Bot	Botryotinia	fuckeliana	NF1		Ascomycota
OY01A01	Abortiporus	biennis	586		Basidiomycota
OY01B01	Abortiporus	biennis	1564		Basidiomycota
OY01C01	Abortiporus	distortus	923	Ps62a	Basidiomycota
OY01D01	Antrodia	albida	561		Basidiomycota
OY01E01	Antrodia	albida	1305	T1d	Basidiomycota
OY01F01	Antrodiella	versicutis, cfr.	581		Basidiomycota
OY01G01	Auriporia	sp	1895		Basidiomycota
OY01H01	Bjerkandera	adusta	538	MAFF-11-20157	Basidiomycota
OY01A02	Bjerkandera	adusta	545		Basidiomycota
OY01B02	Ceriporiopsis	pannocinctus	1781		Basidiomycota
OY01C02	Climacocystis	borealis	8		Basidiomycota
OY01D02	Coriolopsis	glabrorigens	2079		Basidiomycota
OY01E02	Coriolopsis	strumosa	675		Basidiomycota
OY01F02	Coriolopsis	strumosa	1856		Basidiomycota
OY01G02	Cyclomyces	fusca	697		Basidiomycota
OY01H02	Cyclomyces	fusca	1413	Cy1a	Basidiomycota
OY01A03	Cyclomyces	tabacina	599		Basidiomycota
OY01B03	Cyclomyces	tabacina	1539		Basidiomycota
OY01C03	Daedaleopsis	purpurea	1313	T5c	Basidiomycota
OY01D03	Datronia	mollis	635		Basidiomycota
OY01E03	Datronia	mollis	794		Basidiomycota
OY01F03	Datronia	stereoides	1799		Basidiomycota
OY01G03	Diplomitoporus	lindbladii	1906		Basidiomycota
OY01H03	Fomes	fomentarius	556	MAFF-11-20132	Basidiomycota
OY01A04	Fomes	fomentarius	725		Basidiomycota
OY01B04	Fomitella	rhodophaea	606		Basidiomycota
OY01C04	Fomitopsis	feei	1763		Basidiomycota
OY01D04	Fomitopsis	pinicola	197		Basidiomycota
OY01E04	Fomitopsis	pinicola	782	MAFF-11-20130	Basidiomycota
OY01F04	Ganoderma	lucidum	2038		Basidiomycota
OY01G04	Gloeophyllum	subferrugineum	1341	L1b, MAFF-11-20178	Basidiomycota
OY01H04	Gloeophyllum	trabeum	1354		Basidiomycota
OY01A05	Gloeoporus	dichrous	630		Basidiomycota
OY01B05	Grammothele	fuligo	831		Basidiomycota

OY01E05	*Hapalopilus*	*croceus*	1894		Basidiomycota
OY01F05	*Hapalopilus*	*croceus cfr.*	628		Basidiomycota
OY01G05	*Hapalopilus*	*nidulans*	1832		Basidiomycota
OY01H05	*Hexagonia*	*tenuis*	816		Basidiomycota
OY01A06	*Hexagonia*	*tenuis*	1857		Basidiomycota
OY01B06	*Hydnochaete*	*japonica*	921	Ps49a	Basidiomycota
OY01C06	*Hydnochaete*	*japonica*	922	Ps49b	Basidiomycota
OY01D06	*Hydnochaete*	*tabacina*	1557		Basidiomycota
OY01E06	*Hydnochaete*	*tabacinoides*	1652		Basidiomycota
OY01F06	*Inonotus*	*hispidus*	1109	P47a, MAFF-11-20028	Basidiomycota
OY01G06	*Inonotus*	*ludovicianus*	1131	P75a	Basidiomycota
OY01H06	*Inonotus*	*mikadoi*	126		Basidiomycota
OY01A07	*Inonotus*	*sciurinus*	145		Basidiomycota
OY01B07	*Inonotus*	*tomentosa*	165		Basidiomycota
OY01C07	*Irpex*	*lacteus*	656		Basidiomycota
OY01D07	*Irpex*	*lacteus*	670		Basidiomycota
OY01E07	*Ischnoderma*	*resinosum*	1637		Basidiomycota
OY01F07	*Ischnoderma*	*resinosum*	1720		Basidiomycota
OY01G07	*Junghuhnia*	*nitida*	1635		Basidiomycota
OY01H07	*Lenzites*	*betulina*	1987		Basidiomycota
OY01A08	*Melanoporia*	*castanea*	543		Basidiomycota
OY01B08	*Melanoporia*	*castanea*	557		Basidiomycota
OY01C08	*Melanoporia*	*castanea*	724		Basidiomycota
OY01D08	*Meripilus*	*giganteus*	506		Basidiomycota
OY01E08	*Merulius*	*tremellosus*	789		Basidiomycota
OY01F08	*Nigrofomes*	*melanoporus*	604		Basidiomycota
OY01G08	*Nigroporus*	*durus*	1266	F61a	Basidiomycota
OY01H08	*Nigroporus*	*durus*	1267	F61b	Basidiomycota
OY01A09	*Nigroporus*	*vinosus*	1558		Basidiomycota
OY01B09	*Oxyporus*	*cuneatus*	937	Ps80a	Basidiomycota
OY01C09	*Oxyporus*	*populinus, cfr.*	1826		Basidiomycota
OY01D09	*Pachykytospora*	*papyracea*	1701		Basidiomycota
OY01E09	*Pachykytospora*		1676		Basidiomycota
OY01F09	*Paratrichaptum*	*acculatum*	1792		Basidiomycota
OY01G09	*Perenniporia*	*meddulaepanis*	2056		Basidiomycota
OY01H09	*Phaeolus*	*schweiriitzii*	2039		Basidiomycota
OY01A10	*Phaeolus*	*schweinitzii*	2041		Basidiomycota
OY01B10	*Phellinus*	*igniarius*	1149	F3a	Basidiomycota
OY01C10	*Phellinus*	*igniarius*	1150	F3b, MAFF-11-20121	Basidiomycota
OY01D10	*Piptoporus*	*betulinus*	785		Basidiomycota
OY01E10	*Pycnoporellus*	*fulgens*	888	Ps15a	Basidiomycota
OY01F10	*Pycnoporellus*	*fulgens*	889	Ps15b	Basidiomycota
OY01G10	*Pycnoporus*	*cinnabarinus*	773		Basidiomycota
OY01H10	*Pycnoporus*	*cinnabarinus*	1563		Basidiomycota
OY01A11	*Pyrrhoderma*	*sendaiense*	1765		Basidiomycota
OY01B11	*Rigidoporus*	*cinereus*	2094		Basidiomycota
OY01C11	*Rigidoporus*	*microporus*	1575		Basidiomycota
OY01D11	*Rigidoporus*	*microporus*	1601		Basidiomycota
OY01E11	*Schizopora*	*paradoxa*	657		Basidiomycota
OY01F11	*Spongipellis*	*delectans*	2101		Basidiomycota
OY01G11	*Theleporus*	*calcicolor*	1850		Basidiomycota
OY01H11	*Theleporus*	*calcicolor*	1947		Basidiomycota
OY01A12	*Tinctoporellus*	*epimiltinus*	1535		Basidiomycota
OY01B12	*Tinctoporellus*	*epimiltinus*	1941		Basidiomycota
OY01C12	*Tinctoporellus*	sp.	828		Basidiomycota
OY01D12	*Trametes*	*conchifer*	911	Ps41b	Basidiomycota
OY01E12	*Trametes*	*gibbosa*	149		Basidiomycota
OY01F12	*Trametes*	*menzieizii*	1599		Basidiomycota
OY01G12	*Trametes*	*suaveolens*	1320	T14e, MAFF-11-20034	Basidiomycota
OY01H12	*Trechispora*	*mollusca*	1925		Basidiomycota

Table S3. Nematode GH45 sequences obtained in this study

Sequence ID	Labels in the trees	Species	Population/Strain	intron positions	intron length	RT-PCR/RNA-seq
202b	*202b Ruehmaphelenchus sp. NK202*	*Ruehmaphelenchus sp.*	NK202	11	113	
202c	*202c Ruehmaphelenchus sp. NK202*	*Ruehmaphelenchus sp.*	NK202	11	113	
203a	*203a Bursaphelenchus sp. 3 NK203*	*Bursaphelenchus sp. 3*	*NK203*	0	0	
203b	*203b Bursaphelenchus sp. 3 NK203*	*Bursaphelenchus sp. 3*	*NK203*	0	0	
204a	*204a B. doui NK204*	*B. doui*	*NK204*	11	77	yes
204b	*204b B. doui NK204*	*B. doui*	*NK204*	11	40	yes
204c	*204c B. doui NK204*	*B. doui*	*NK204*	11	78	yes
204d	*204d B. doui NK204*	*B. doui*	*NK204*	11	42	yes
204e	*204e B. doui NK204*	*B. doui*	*NK204*	11	98	yes
205a	*205a B. luxuriosiae NK205*	*B. luxuriosiae*	*NK205*	11	220	
205c	*205c B. luxuriosiae NK205*	*B. luxuriosiae*	*NK205*	0	0	
205d	*205d B. luxuriosiae NK205*	*B. luxuriosiae*	*NK205*	11	87	
206a	*206a Bursaphelenchus sp. 1 NK206*	*Bursaphelenchus sp. 1*	*NK206*	11	80	
206b	*206b Bursaphelenchus sp. 1 NK206*	*Bursaphelenchus sp. 1*	*NK206*	11	80	
207b	*207b Bursaphelenchus sp. 3 NK207*	*Bursaphelenchus sp. 3*	NK207	0	0	
207d	*207d Bursaphelenchus sp. 3 NK207*	*Bursaphelenchus sp. 3*	NK207	0	0	
209b	*209b B. yongensis NK209*	*B. yongensis*	NK209	0	0	
209c	*209c B. yongensis NK209*	*B. yongensis*	NK209	0	0	
210b	*210b Bursaphelenchus sp. 2 NK210*	*Bursaphelenchus sp.2*	NK210	0	0	
212a	*212a B. okinawensis NK212*	*B. okinawensis*	NK212	0	0	
212b	*212b B. okinawensis NK212*	*B. okinawensis*	NK212	0	0	
212d	*212d B. okinawensis NK212*	*B. okinawensis*	NK212	0	0	
212e	*212e B. okinawensis NK212*	*B. okinawensis*	NK212	0	0	
215a	*215a B. kiyoharai NK215*	*B. kiyoharai*	*NK215*	11	38	
216a	*216a B. purviscularis NK216*	*B. purviscularis*	*NK216*	11	79	
216c	*216c B. purviscularis NK216*	*B. purviscularis*	*NK216*	11	91	
216e	*216e B. purviscularis NK216*	*B. purviscularis*	*NK216*	11	112	
216f	*216f B. purviscularis NK216*	*B. purviscularis*	*NK216*	0	0	
217a	*217a B. doui NK217*	*B. doui*	*NK217*	11	78	
217c	*217c B. doui NK217*	*B. doui*	*NK217*	11	97	
217d	*217d B. doui NK217*	*B. doui*	*NK217*	11	40	
220c	*220c Bursaphelenchus sp. 3 NK220*	*Bursaphelenchus sp. 3*	*NK220*	0	0	
220d	*220d Bursaphelenchus sp. 3 NK220*	*Bursaphelenchus sp. 3*	*NK220*	0	0	
220e	*220e Bursaphelenchus sp. 3 NK220*	*Bursaphelenchus sp. 3*	*NK220*	0	0	
221a	*221a B. kiyoharai NK221*	*B. kiyoharai*	NK221	11	38	
225b	*225b B. poligraphi NK225*	*B. poligraphi*	NK225	0	0	
Bc_cel45A	*Bc_cel45A B. conicaudatus NKBc*	*B. conicaudatus*	NKBC	11	36	
Bc_cel45B	*Bc_cel45B B. conicaudatus NKBc*	*B. conicaudatus*	NKBC	11	36	
Bc_cel45E	*Bc_cel45E B. conicaudatus NKBc*	*B. conicaudatus*	NKBC	11	36	
Bd_cel45D	*Bd_cel45D B. doui NK217*	*B. doui*	NK217	0	0	
Bl_cel45D	*Bl_cel45D B. luxuriosiae NARA*	*B. luxuriosiae*	NARA	11	79	
Bm_eng1	*Bm_eng1 B. mucronatus Un1*	*B. mucronatus*	Un1	0	0	
Bm_cel45A	*Bm_cel45A B. mucronatus Un1*	*B. mucronatus*	Un1	11	66	yes
Bm_cel45B	*Bm_cel45B B. mucronatus Un1*	*B. mucronatus*	Un1	11	66	yes
Bx_eng_2	*Bx_eng2 B. xylophilus Ka4c1*	*B. xylophilus*	Ka4C1	11	133	yes
Bx_eng_3	*Bx_eng3 B. xylophilus Ka4c1*	*B. xylophilus*	Ka4C1	11	97	yes
Bx_eng_1	*Bx_eng1 B. xylophilus Ka4c1*	*B. xylophilus*	Ka4C1	0	0	yes
s00119.44	*s00119.44 B. xylophilus Ka4c1*	*B. xylophilus*	Ka4C1	0	0	
s00397.15	*s00397.15 B. xylophilus Ka4c1*	*B. xylophilus*	Ka4C1	8	356	yes
s00397.16	*s00397.16 B. xylophilus Ka4c1*	*B. xylophilus*	Ka4C1	8	356	yes
s00397.6	*s00397.6 B. xylophilus Ka4c1*	*B. xylophilus*	Ka4C1	8	182	yes
s01038.221	*s01038.221 B. xylophilus_Ka4c1*	*B. xylophilus*	Ka4C1	0	0	
s01116.1	*s01116.1 B. xylophilus Ka4c1*	*B. xylophilus*	Ka4C1	0	0	
s01147.110	*s01147.110 B. xylophilus Ka4c1*	*B. xylophilus*	Ka4C1	0	0	
s01288.37	*s01288.37 B. xylophilus Ka4c1*	*B. xylophilus*	Ka4C1	0	0	
A_BESSEYI	*Aphelenchoides besseyi*	*Aphelenchoides besseyi*	-	-	-	

Table S4. Fungal GH45 sequences obtained in this study

Sequence ID	Label in the trees	Species	position introns	length introns
01A04a	01A04a_Coniothyrium_sp	*Coniothyrium* sp.	6	53
01A07a	01A07a_Coniochaeta_sp	*Coniochaeta* sp.	6	59
01A08c	01A08c_Dothidea_sp	*Dothidea* sp.	5,6	64,52
01A10a	01A10a_Fusarium_oxysporum	*Fusarim oxysporum*	6	50
01A12a	01A12a_Hendersonia_sp	*Hendersonia* sp.	6	56
01B01a	01B01a_Alternaria_tenuis	*Alternaria tenuis*	6	63
01B04b	01B04b_Coniothyrium_concentricum	*Coniothyrium concentricum*	6	71
01B06a	01B06a_Chloroscypha_chamaecyparidis	*Chloroscypha chamaecyparidis*	1,11	43,53
01B09a	01B09a_Epicoccum_sp	*Epicoccum sp.*	6	52
01B10a	01B10a_Fusarium_lateritium	*Fusarium lateritium*	6	56
01B11a	01B11a_Glomerella_sp	*Glomerella* sp.	11	343
01C05a	01C05a_Corynespora_cassiicola	*Corynespora cassiicola*	6	54
01C06a	01C06a_Cryptosporiopsis_sp	*Cryptosporiopsis* sp.	11	80
01C09a	01C09a_Epicoccum_sp	*Epicoccum* sp.	6	52
01C11a	01C11a_Gliocladium_sp	*Gliocladium* sp,	6	62
01D03a	01D03a_Colletotrichum_truncatum	*Colletotrichum truncatum*	6,10	54,53
01D05a	01D05a_Cylindrosporium_sp	*Cylindrosporium* sp.	0	0
01E01b	01E01b_Ascochyta_pisi	*Ascochyta pisi*	6	52
01E02b	01E02b_Botryosphaeria_sp	*Botryosphaeria* sp.	6	75
01E02c	01E02c_Botryosphaeria_sp	*Botryosphaeria* sp.	6	98
01E04a	01E04a_Davidiella_sp	*Davidiella* sp.	6	51
01E11a	01E11a_Pyrenochaeta_sp	*Pyrenochaeta* sp.	6	82
01F02a	01F02a_Xenostigmina_sp	*Xenostigmina* sp.	6	112
01F08a	01F08a_Leptosphaerulina_sp	*Leptosphaerulina* sp.	6	50
01F11a	01F11a_Helminthosporium_oryzae	*Helminthosporium oryzae*	6	52
01F11b	01F11b_Helminthosporium_oryzae	*Helminthosporium oryzae*	6	56
01F12a	01F12a_Pestalotiopsis_sp	*Pestalotiopsis* sp.	0	0
01F12b	01F12b_Pestalotiopsis_sp	*Pestalotiopsis* sp.	2,6	184, 71
01G04a	01G04a_Curvularia_lunata	*Curvularia lunata*	6	56
01H03a	01H03a_Calonectria_kyotensis	*Calonectria kyotensis*	6	54
02A04c	02A04c_Nectria_cinnabarina	*Nectria cinnabarina*	6	54
02A05a	02A05a_Pestalotia_aceris	*Pestalotia aceris*	0	0
02A07a	02A07a_Septotinia_populiperda	*Septotinia populiperda*	11	70
02A12a	02A12a_Bartalinia_robillardoides	*Bartalinia robillardoides*	6	56
02A12b	02A12b_Bartalinia_robillardoides	*Bartalinia robillardoides*	11	58
02B03a	02B03a_Macrophomina_phaseolina	*Macrophomina phaseolina*	6	59
02B04a	02B04a_Nectria_sp	*Nectria* sp.	6	54
02B07b	02B07b_Septotis_populiperda	*Septotis populiperda*	11	70
02B08c	02B08c_Stagonospora_cinnamomum	*Stagonospora cinnamomum*	6	51
02C02a	02C02a_Monochaetia_monochaeta	*Monochaetia monochaeta*	11	119
02C02b	02C02b_Monochaetia_monochaeta	*Monochaetia monochaeta*	6	70
02C04a	02C04a_Neocosmospora_sp	*Neocosmospora sp.*	6	51
02C05a	02C05a_Epicoccum_sp	*Epicoccum* sp.	6	52
02C06a	02C06a_Melanomma_sp	*Melanomma* sp.	6	89
02C09a	02C09a_Truncatella_sp	*Truncatella* sp.	6	50
02C10a	02C10a_Paraphoma_sp	*Paraphoma* sp.	6	52
02C11a	02C11a_Chaetomium_sp	*Chaetomium* sp.	11	69
02D05a	02D05a_Phyllosticta_alcides	*Phyllosticta alcides*	6	53
02D06a	02D06a_Rhizina_undulata	*Rhizina undulata*	3,7,12	58,66,56
02D07a	02D07a_Sclerotinia_sclerotiorum	*Sclerotinia sclerotiorum*	9	74
02E02a	02E02a_Macrosporium_sp	*Macrosporium* sp.	6	63
02E05a	02E05a_Patellina_sp	*Patellina* sp.	11	144
02E05b	02E05b_Patellina_sp	*Patellina* sp.	11	48
02E07a	02E07a_Sclerotinia_camelliae	*Sclerotinia camelliae*	11	92
02E08a	02E08a_Scolicosporium_sp	Scolicosporium sp.	6	63
02E12a	02E12a_Ascocalyx_pinicola	*Ascocalyx pinicola*	4,11	89,86
02F02a	02F02a_Metasphaeria_sp	*Metasphaeria* sp.	6	67
02F05a	02F05a_Pestalotiopsis_foedans	*Pestalotiopsis foedans*	0	0
02F08a	02F08a_Tubercularia_vulgaris	*Tubercularia vulgaris*	6	54
02G04a	02G04a_Pestalotiopsis_guepini	*Pestalotiopsis guepinii*	0	0
02G09a	02G09a_Pyrenochaeta_cava	*Pyrenochaeta cava*	6	82
02H03a	02H03a_Mycosphaerella_sojae	*Mycosphaerella sojae*	6	50
02H05a	02H05a_Pestalotiopsis_glandicola	*Pestalotiopsis glandicola*	0	0
02H05b	02H05b_Pestalotiopsis_glandicola	*Pestalotiopsis glandicola*	2,6	223,75
02H08a	02H08a_Tuberculis_sp	*Tuberculis* sp.	0	0
Botcel45A	Botcel45A_Botryotinia_fuckeliana	*Botryotinia fuckeliana*	9	76
Botcel45B	Botcel45B_Botryotinia_fuckeliana	*Botryotinia fuckeliana*	11	68
Botcel45C	Botcel45C_Botryotinia_fuckeliana	*Botryotinia fuckeliana*	0	0
01G12_L52a	01G12_L52a_Lachnellula_sp	*Lachnellula* sp.	11	46
CAP61565	CAP61565_Podospora_anserina	*Podospora anserina*	6	53
CAP694443	CAP694443_Podospora_anserina	*Podospora anserina*	0	0
CBX93072	CBX93072_Leptosphaeria_maculans	*Leptosphaeria maculans*	11	53
CBX97547	CBX97547_Leptosphaeria maculans	*Leptosphaeria maculans*	6	53
CCD33730	CCD33730_Botryotinia_fuckeliana	*Botryotinia fuckeliana*	9	76
CCD48539	CCD48539_Botryotinia_fuckeliana	*Botryotinia fuckeliana*	11	68

HM003039	HM003039_Phialophora_sp	*Phialophora* sp.	-	-
GU372728	GU372728_Rhizoctonia_solani	*Rhizoctonia solani*	-	-
GU372729	GU372729_Rhizoctonia_solani	*Rhizoctonia solani*	-	-
AB175926	AB175926_Phycomyces_nitens	*Phycomyces nitens*	-	-
A68060	A68060_Humicola_insolence	*Humicola insolence*	-	-
AB003108	AB003108_Humicola_grisea	*Humicola grisea*	-	-
AB047927	AB047927_Rhizopus_oryzae	*Rhizopus oryzae*	-	-
AB056667	AB056667_Rhizopus_oryzae	*Rhizopus oryzae*	-	-
AB056668	AB056668_Rhizopus_oriza	*Rhizopus oryzae*	-	-
AB175927	AB175927_Mucor_circinelloides	*Mucor circinelloides*	-	-
AB175928	AB175928_Mucor_circinelloides	*Mucor circinelloides*	-	-
AB248917	AB248917_Staphylotrichum_coccosporum	*Staphylotrichum coccosporum*	-	-
AF176572	AF176572_Alternaria_alternata	*Alternaria alternata*	-	-
AJ515703	AJ515703_Melanocarpus_albomyces	*Melanocarpus albomyces*	11	-
AY342397	AY342397_Gibberella_zeae	*Gibberella zeae*	-	-
CAD70529	CAD70529_Neurospora_cras	*Neurospora crassa*	-	-
CS256106	CS256106_Talaromyces_emersonii	*Talaromyces emersonii*	-	-
EU057152	EU057152_Syncephalastrum_racemosum	*Syncephalastrum racemosum*	-	-
FSOKCH	FSOKCH_Fusarium_oxysporum	*Fusarium oxysporum*	-	-
S81598	S81598_Ustilago_maydis	*Ustilago maydis*	-	-

Table S5. Sequences and Genbank accession numbers used in Figure 4

Species	Sequence ID	GenBank accession number
Komagataella pastoris		JQ689069
Mucor racemosus		AJ271061
Phycomyces sp.		DQ273800
Ustilago sp.		AF453938
Neurospora sp.		AY681158
Humicola sp.		DQ237875
Fusarium oxysporum		EF590327
Talaromyces sp.		DQ010015
Coniothyrium sp.	01A04	KF590112
Coniocaeta sp.	01A07	KF590113
Dothidea sp.	01A08	KF590114
Fusarium oxysporum	01A10	KF590115
Hendersonia sp.	01A12	KF590116
Alternaria tenuis	01B01	KF590117
Coniothyrium concentricum	01B04	KF590118
Chlorosrypha chamaecyparidis	01B06	KF590119
Epicoccum sp.	01B09	KF590120
Fusarium lateritium	01B10	KF590121
Glomerella ochraeum	01B11	KF590122
Corynespora cassiicola	01C05	KF590123
Crypotosporiopsis sp.	01C06	KF590124
Epicoccum sp.	01C09	KF590125
Gliocladium sp.	01C11	KF590126
Colletotrichum truncatum	01D03	KF590127
Cylindrosporium sp.	01D05	KF590128
Ascochyta pisi	01E01	KF590129
Botryosphaeria sp.	01E02	KF590130
Davidiella sp.	01E04	KF590131
Pyrenochaeta sp.	01E11	KF590132
Xenostigmina sp.	01F02	KF590133
Leptosphaerulina sp.	01F08	KF590134
Helminthosporium oryzae	01F11	KF590135
Pestalotiopsis sp.	01F12	KF590136
Curvularia lunata	01G04	KF590137
Lachnellula sp.	01G12	KF590138
Cylindrocladium kyotensis	01H03	KF590139
Nectria cinnabarina	02A04	KF590140
Pestalotia aceris	02A05	KF590141
Septotinia populiperda	02A07	KF590142
Bartalinia robillardoides	02A12	KF590143
Macrophomina phaseolina	02B03	KF590144
Nectria sp.	02B04	KF590145
Septotis populiperda	02B07	KF590146
Stagonospora cinnamomum	02B08	KF590147
Monochaetia monochaeta	02C02	KF590148
Neocosmospora sp.	02C04	KF590149
Epicoccum sp.	02C05	KF590150
Melanomma sp.	02C06	KF590151
Truncatella sp.	02C09	KF590152
Paraphoma sp.	02C10	KF590153
Chaetomium sp.	02C11	KF590154
Phyllosticta alcides	02D05	KF590155

Sclerotinia sclerotiorum	02D07	KF590157
Macrosporium sp.	02E02	KF590158
Patellina sp.	02E05	KF590159
Sclerotinia camelliae	02E07	KF590160
Scolicosporium sp.	02E08	KF590161
Ascocalyx pinicola	02E12	KF590162
Metasphaeria sp.	02F02	KF590163
Pestalotiopsis foedans	02F05	KF590164
Tubercularia vulgaris	02F08	KF590165
Pestalotiopsis guepini	02G04	KF590166
Pyrenochaeta sp.	02G09	KF590167
Mycosphaerella sojae	02H03	KF590168
Pestalotiopsis glandicola	02H05	KF590169
Tuberculis sp.	02H08	KF590170
Botryotinia fuckeliana		AY544651
Leptosphaeria maculans		JF740307
Melanocarpus albomyces		JQ067902
Phialophora livistonae		KC005796
Podospora anserina		FR774293
Rhizoctonia solani		JX576188
Rhizopus oryzae		JX961692
Staphylotrichum coccosporum		AB625573
Alternaria alternata		DQ678082
Fusarium asiaticum		AB586991
Syncephalastrum racemosum		JX961686
Humicola insolens		EU257375

Table S6. Species names and GenBank accession numbers used in Figure 6

Species	Sequence ID	GenBank accession number
Ruehmaphelenchus sp.	202b	KF590171
Ruehmaphelenchus sp.	202c	KF590172
B. niphades	203a	KF590173
B. niphades	203b	KF590174
B. doui	204a	KF590175
B. doui	204b	KF590176
B. doui	204c	KF590177
B. doui	204d	KF590178
B. doui	204e	KF590179
B. luxuriosiae	205a	KF590180
B. luxuriosiae	205c	KF590181
B. luxuriosiae	205d	KF590182
Bursaphelenchus sp. 1	206a	KF590183
Bursaphelenchus sp. 1	206b	KF590184
B. niphades	207b	KF590185
B. niphades	207d	KF590186
B. yongensis	209b	KF590187
B. yongensis	209c	KF590188
Bursaphelenchus sp.2	210b	KF590189
B. okinawensis	212a	KF590190
B. okinawensis	212b	KF590191
B. okinawensis	212d	KF590192
B. okinawensis	212e	KF590193
B. kiyoharai	215a	KF590194
B. purviscularis	216a	KF590195
B. purviscularis	216c	KF590196
B. purviscularis	216e	KF590197
B. purviscularis	216f	KF590198
B. doui	217a	KF590199
B. doui	217c	KF590200
B. doui	217d	KF590201
B. niphades	220c	KF590202
B. niphades	220d	KF590203
B. niphades	220e	KF590204
B. kiyoharai	221a	KF590205
B. poligraphi	225b	KF590206
B. conicaudatus	Bc_cel45A	KF590207
B. conicaudatus	Bc_cel45B	KF590208
B. conicaudatus	Bc_cel45E	KF590209
B. doui	Bd_cel45D	KF590210
B. luxuriosiae	Bl_cel45D	KF590211
B. mucronatus	Bm_eng1	KF590213
B. mucronatus	Bm_cel45A	KF590214
B. mucronatus	Bm_cel45B	KF590212
Bx_eng_1_sp0	Bx_eng_1	BAD34544
Bx_eng_2_sp5	Bx_eng_2	BAD34546
Bx_eng_3_sp5	Bx_eng_3	BAD34548
Aphelenchodies besseyi	A_BESSEYI	(EST)
Coniothyrium sp.	01A04a	KF590043
Coniochaeta sp.	01A07a	KF590044
Dothidea sp.	01A08c	KF590045
Fusarim oxysporum	01A10a	KF590046
Hendersonia sp.	01A12a	KF590047
Alternaria tenuis	01B01a	KF590048
Coniothyrium concentricum	01B04b	KF590049
Chloroscypha chamaecyparidis	01B06a	KF590050
Epicoccum sp.	01B09a	KF590051
Fusarium lateritium	01B10a	KF590052
Glomerella sp.	01B11a	KF590053
Corynespora cassiicola	01C05a	KF590054
Cryptosporiopsis sp.	01C06a	KF590055
Epicoccum sp.	01C09a	KF590056
Gliocladium sp.	01C11a	KF590057
Colletotrichum truncatum	01D03a	KF590058
Cylindrosporim sp.	01D05a	KF590059

Botryosphaeria sp.	01E02c	KF590062
Davidiella sp.	01E04a	KF590063
Pyrenochaeta sp.	01E11a	KF590064
Xenostigmina sp.	01F02a	KF590065
Leptosphaerulina sp.	01F08a	KF590066
Helminthosporium oryzae	01F11a	KF590067
Helminthosporium oryzae	01F11b	KF590068
Pestalotiopsis sp.	01F12a	KF590069
Pestalotiopsis sp.	01F12b	KF590070
Curvularia lunata	01G04a	KF590071
Calonectria kyotensis	01H03a	KF590072
Nectria cinnabarina	02A04c	KF590073
Pestalotia aceris	02A05a	KF590074
Septotinia populiperda	02A07a	KF590075
Bartalinia robillardoides	02A12a	KF590076
Bartalinia robillardoides	02A12b	KF590077
Macrophomina phaseolina	02B03a	KF590078
Nectria sp.	02B04a	KF590079
Septotis populiperda	02B07b	KF590080
Stagonospora cinnamomum	02B08c	KF590081
Monochaetia monochaeta	02C02a	KF590082
Monochaetia monochaeta	02C02b	KF590083
Neocosmospora sp.	02C04a	KF590084
Epicoccum sp.	02C05a	KF590085
Melanomma sp.	02C06a	KF590086
Truncatella sp.	02C09a	KF590087
Paraphoma sp.	02C10a	KF590088
Chaetomium sp.	02C11a	KF590089
Phyllosticta alcides	02D05a	KF590090
Rhizina undulata	02D06a	KF590091
Sclerotinia sclerotiorum	02D07a	KF590092
Macrosporium sp.	02E02a	KF590093
Patellina sp.	02E05a	KF590094
Patellina sp.	02E05b	KF590095
Sclerotinia camelliae	02E07a	KF590096
Scolicosporium sp.	02E08a	KF590097
Ascocalyx pinicola	02E12a	KF590098
Metasphaeria sp.	02F02a	KF590099
Pestalotiopsis foedans	02F05a	KF590100
Tubercularia vulgaris	02F08a	KF590101
Pestalotiopsis guepini	02G04a	KF590102
Pyrenochaeta cava	02G09a	KF590103
Mycosphaerella sojae	02H03a	KF590104
Pestalotiopsis glandicola	02H05a	KF590105
Pestalotiopsis glandicola	02H05b	KF590106
Tuberculis sp.	02H08a	KF590107
Botryotinia fuckeliana	Botcel45A	KF590108
Botryotinia fuckeliana	Botcel45B	KF590109
Botryotinia fuckeliana	Botcel45C	KF590110
Lachnellula sp.	01G12_L52a	KF590111
Apriona germari		AAU44973
Apriona germari		AAN78326
Anoplophora chinensis		AFN89565
Oncideres albomarginata		ADI24132
Apriona germari		AAR22385
Phaedon cochleariae		CCJ09450
Leptinotarsa decemlineata		ADU33350
Phaedon cochleariae		CAA76931
Leptinotarsa decemlineata		ADU33351
Leptinotarsa decemlineata		ADU33348
Phaedon cochleariae		CCJ09451
Chrysomela tremula		ADU33285
Sitophilus oryzae		ADU33246
Leptinotarsa decemlineata		ADU33349
Diabrotica virgifera		AFI56547
Reticulitermes speratus _hindgut_protist_130484		BAA98031
Reticulitermes speratus _hindgut_protist_130485		BAA98037

Reticulitermes speratus _hindgut_protist_130488	BAA98030
Reticulitermes speratus _hindgut_protist_130489	BAA98029
Reticulitermes speratus _hindgut_protist_130490	BAA98039
Reticulitermes speratus _hindgut_protist_130491	BAA98040
Reticulitermes speratus _hindgut_protist_130492	BAA98035
Reticulitermes speratus _hindgut_protist_130493	BAA98047
Reticulitermes speratus _hindgut_protist_130494	BAA98046
Reticulitermes speratus _hindgut_protist_130495	BAA98045
Reticulitermes speratus _hindgut_protist_130496	BAA98044
Reticulitermes speratus _hindgut_protist_130497	BAA98032
Reticulitermes speratus _hindgut_protist_130498	BAA98043
uncultured_symbiotic_protist_of_*Reticulitermes_speratus*	BAF57325
uncultured_symbiotic_protist_of_*Reticulitermes_speratus*	BAF57323
Reticulitermes speratus _hindgut_protist_130498	BAA98042
uncultured_symbiotic_protist_of_*Reticulitermes_speratus*	BAF57324
eukaryotic_synthetic_construct	BAG71490
Reticulitermes_speratus_hindgut_protist_130484	BAA98049
uncultured_symbiotic_protist_of_*Hodotermopsis_sjoestedti*	BAF57357
Reticulitermes_speratus _hindgut_protist_130484	BAA98034
Reticulitermes_speratus _hindgut_protist_130485	BAA98048
uncultured_symbiotic_protist_of_*Hodotermopsis_sjoestedti*	BAF57359
uncultured_symbiotic_protist_of_*Hodotermopsis_sjoestedti*	BAF57356
uncultured_symbiotic_protist_of_*Hodotermopsis_sjoestedti*	BAF57355
uncultured_symbiotic_protist_of_*Cryptocercus_punctulatus*	BAF57479
Mastotermes_darwiniensis _hindgut_symbiont	CAD39200
uncultured_symbiotic_protist_of_*Mastotermes_darwiniensis*	BAF57444
uncultured_symbiotic_protist_of_*Mastotermes_darwiniensis*	BAF57451
Mastotermes_darwiniensis _hindgut_symbiont	CAD39199
uncultured_symbiotic_protist_of_Mastotermes_darwiniensis	BAF57441
Mastotermes_darwiniensis _hindgut_symbiont_sp	CAD39197
Mastotermes_darwiniensis _hindgut_symbiont	CAD39198
uncultured_symbiotic_protist_of_*Mastotermes_darwiniensis*	BAF57454
uncultured_symbiotic_protist_of_*Mastotermes_darwiniensis*	BAF57452
uncultured_symbiotic_protist_of_*Mastotermes_darwiniensis*	BAF57447
uncultured_symbiotic_protist_of_*Mastotermes_darwiniensis*	BAF57455
uncultured_symbiotic_protist_of_*Mastotermes_darwiniensis*	BAF57445
uncultured_symbiotic_protist_of_*Mastotermes_darwiniensis*	BAF57446
uncultured_symbiotic_protist_of_*Mastotermes_darwiniensis*	BAF57442
uncultured_symbiotic_protist_of_*Mastotermes_darwiniensis*	BAF57450
uncultured_symbiotic_protist_of_*Mastotermes_darwiniensis*	BAF57453
uncultured_symbiotic_protist_of_*Hodotermopsis_sjoestedti*	BAF57360
uncultured_symbiotic_protist_of_*Hodotermopsis_sjoestedti*	BAF57355
Rhizoctonia solani	ADV02787
Rhizoctonia solani	ADV02788
Botryotinia fuckeliana	CCD33730
Botryotinia fuckeliana	CCD48539
Humicola grisea	BAA74956
Komagataella pastoris	CCA40496
Komagataella pastoris	CAY71902
Leptosphaeria maculans	CBX93072
Leptosphaeria maculans	CBX97547
Phialophora sp.	ADZ99360
Rhizopus stolonifer	ADV02787
Rhizopus stolonifer	ADV02788
Alternaria alternata	AAF05700
Gibberella zeae	AAR02399
Syncephalastrum racemosum	ABU49185
Humicola grisea	BAA74957
Rhizopus oryzae	BAC53956
Rhizopus oryzae	BAC53987
Rhizopus oryzae	BAC53988
Phycomyces nitens	BAD77808
Mucor circinelloides	BAD95808
Mucor circinelloides	BAD95809
Melanocarpus albomyces	CAD56665
Neurospora crassa	CAD70529
Talaromyces emersonii	CAJ75963

Ustilago maydis	XP762479
Podospora anserina	CAP69443
Podospora anserina	CAP61565
Staphylotrichum coccosporum	BAG69187
