# Supplementary material for: Distribution and evolution of glycoside hydrolase family 45 cellulases in nematodes and fungi
Source: BMC Evol Biol. 2014 Apr 1;14:69. doi: 10.1186/1471-2148-14-69 (PMC3997829; doi:10.1186/1471-2148-14-69)
Supplement: Additional file 2: Figure S1 — Amino acid alignment of GH45 proteins and intron positions. Intron positions are indicated by triangles on the alignment. Conserved regions used to design primers are boxed. Asterisks indicate the two catalytic core residues (Asp, Asp). The numbers to the left indicate the amino acid position of the respective proteins. Phase of the introns is shown by distinct triangles. BxENG1, 2 and 3 – B. xylophilus sequences (nematode – BAD34543-5), B_ciner1 – GHF45 cellulase from Botrytis cinerea (fungus – CCD33730), R.oryza1 – Rhizopus oryzae (fungus – BAC53956), H_insol1 – Humicola insolens (fungus – CAB42307), A.germ1 – Apriona germari (insect – AAN78326). Figure S2. Bayesian 50% majority rule consensus tree of GH45 amino acid sequences from Aphelenchoidea under WAG + I + G + F model. Posterior probabilities more than 65% are given for appropriate clades; bootstrap values greater than 50% are given on appropriate clades in ML analysis. Figure S3. Bayesian 50% majority rule consensus tree of GH45 amino acid sequences from Ascomycota, Basidiomycota and Zygomycota under LG + I + G model. Posterior probabilities more than 65% are given for appropriate clades; bootstrap values greater than 50% are given on appropriate clades in ML analysis. [file 1471-2148-14-69-S2.pdf]

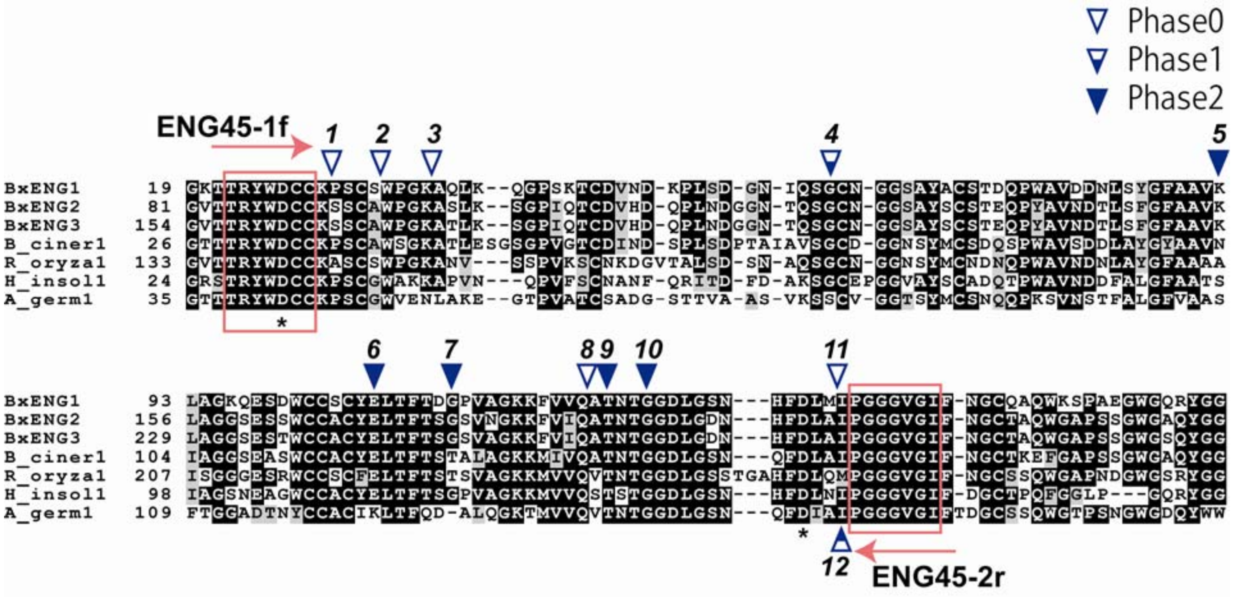

Figure S1. Amino acid alignment of GH45 proteins and intron positions. Intron positions are indicated by triangles on the alignment. Conserved regions used to design primers are boxed. Asterisks indicate the two catalytic core residues (Asp, Asp). The numbers to the left indicate the amino acid position of the respective proteins. Phase of the introns is shown by distinct triangles. BxENG1, 2 and 3 – *B. xylophilus* sequences (nematode – BAD34543-5), B_ciner1 – GHF45 cellulase from *Botrytis cinerea* (fungus – CCD33730), R.oryza1 – *Rhizopus oryzae* (fungus – BAC53956), H_insol1 – *Humicola insolens* (fungus – CAB42307), A.germ1 – *Apriona germari* (insect – AAN78326).

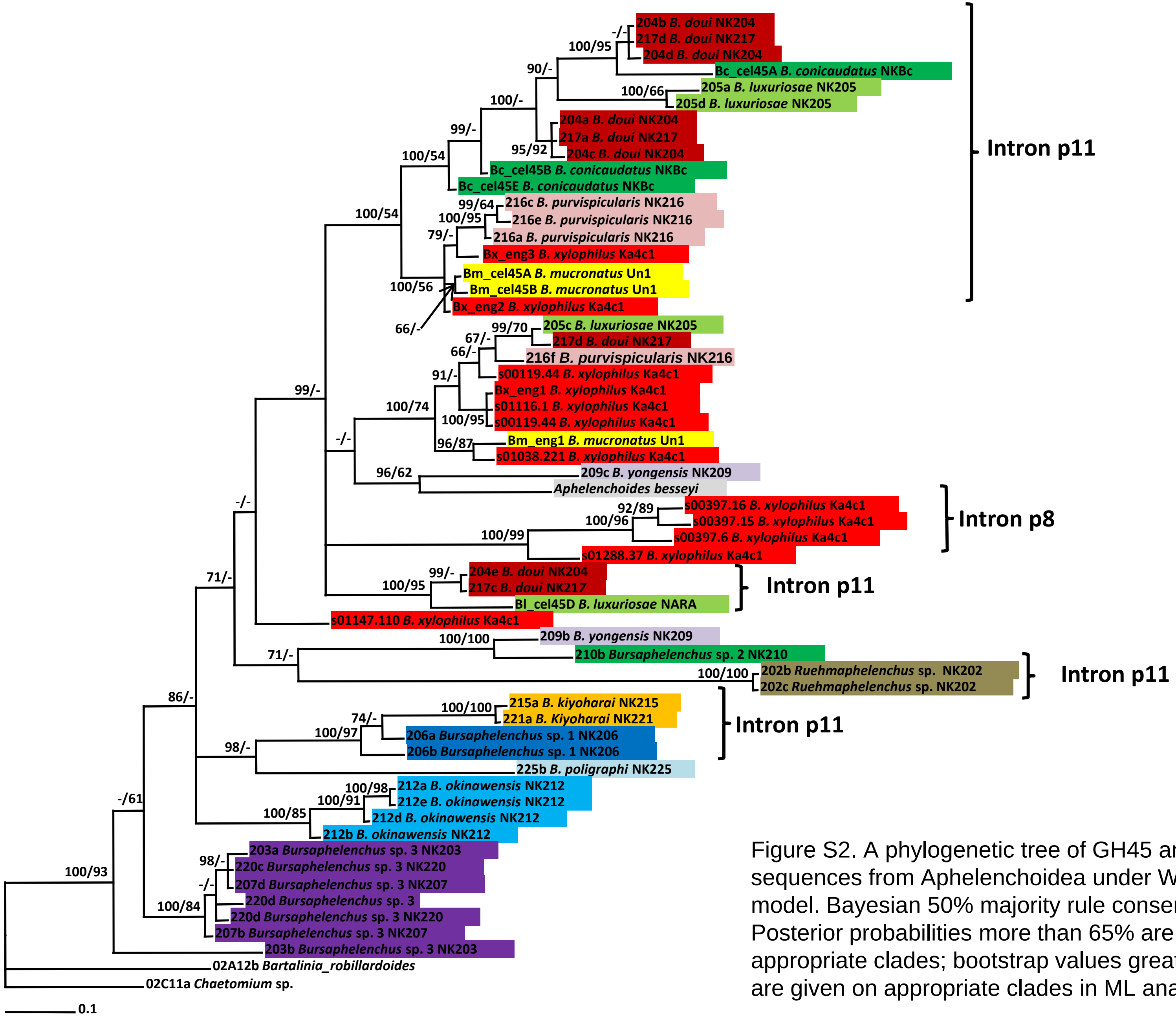

Figure S2. A phylogenetic tree of GH45 amino acid sequences from Aphelenchoidea under WAG+I+G+F model. Bayesian 50% majority rule consensus tree. Posterior probabilities more than 65% are given for appropriate clades; bootstrap values greater than 50% are given on appropriate clades in ML analysis.

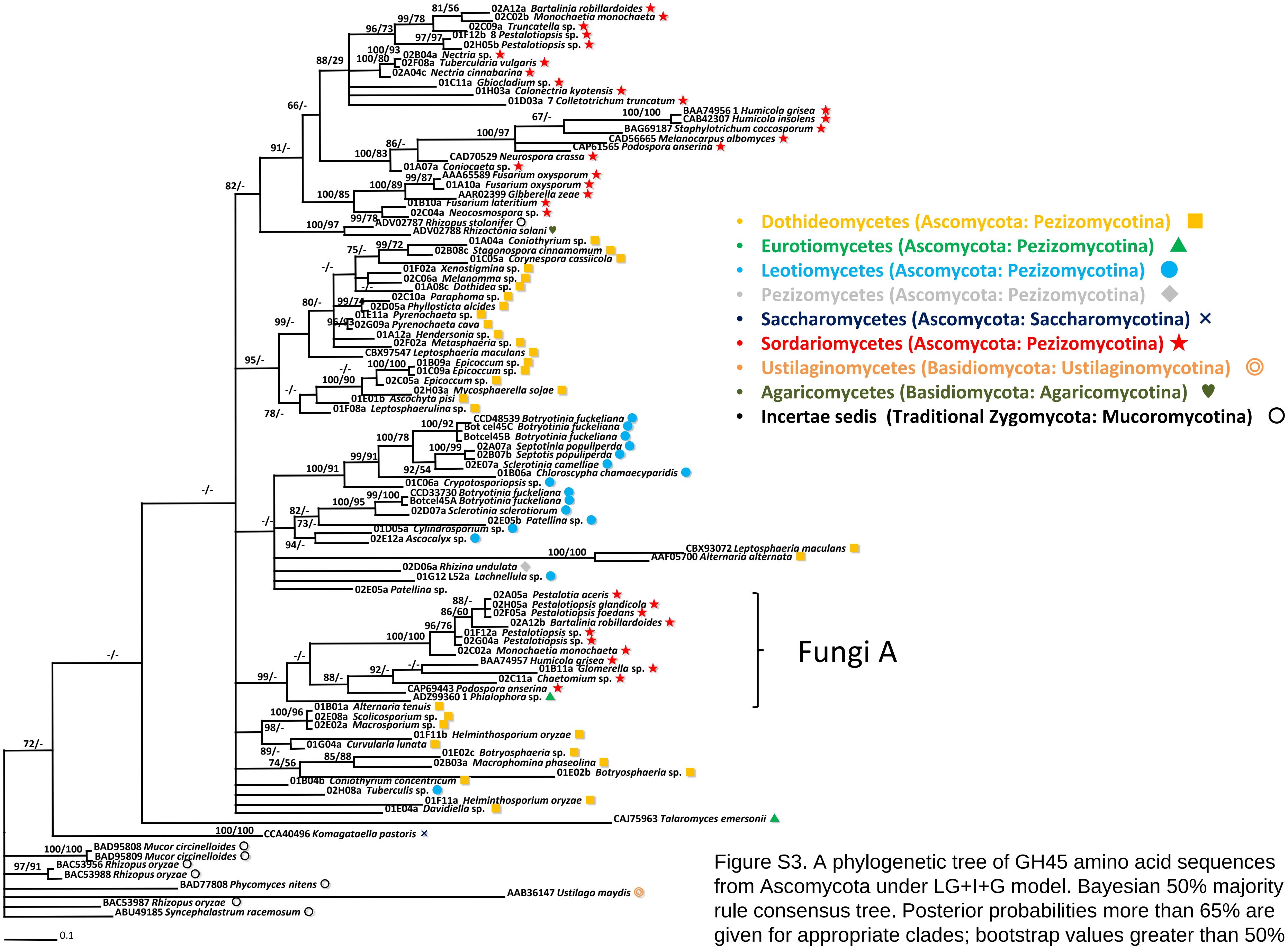

Figure S3. A phylogenetic tree of GH45 amino acid sequences from Ascomycota under LG+I+G model. Bayesian 50% majority rule consensus tree. Posterior probabilities more than 65% are given for appropriate clades; bootstrap values greater than 50% are given on appropriate clades in ML analysis.
